# Outcomes of an Evidence-Based Telemental Health Program Across Sexual Orientation and Gender Identity

**DOI:** 10.1177/24731242251382353

**Published:** 2025-10-06

**Authors:** Jocelynn T. Owusu, Katherine B. Shakman, Keren Lehavot, Robert E. Wickham, Alethea A. Varra, Connie Chen, Anita Lungu, Jennifer L. Lee

**Affiliations:** ^1^ Lyra Health, Burlingame, California, USA.; ^2^ Department of Psychological Sciences, Northern Arizona University, Flagstaff, Arizona, USA.

**Keywords:** depression, anxiety, mental health, telehealth, digital health, sexual and gender minorities

## Abstract

**Introduction:**

The effectiveness of evidence-based mental health programs across sexual orientation and minoritized gender identity groups is not well-understood.

**Objectives:**

To evaluate outcomes of an employer-offered, evidence-based telemental health program (video-based psychotherapy sessions supplemented by asynchronous, guided practice sessions) across gender identity and sexual orientation groups.

**Methods:**

This retrospective cohort analysis included 29,860 U.S.-based adults with clinically elevated anxiety and/or depression symptoms, who began a culturally responsive, telemental health program between April 2021 and April 2023. The primary outcomes were changes in anxiety (Generalized Anxiety Disorder-7) and depression symptoms (Patient Health Questionnaire-9) during, and at the end of, care.

**Results:**

Participants self-identified as female (63.7%), male (31.5%), and transgender and gender diverse (1.4%); participants also self-identified as bisexual (5.5%), gay or lesbian (4.1%), additional sexual orientation groups (5.4%), and straight (78.0%). Rates of end-of-care reliable improvement or recovery in anxiety or depression symptoms ranged from 82.4 − 87.5% across gender identity groups, and 84.3 − 86.9% across sexual orientation groups. In growth curve models, anxiety and depression symptoms significantly decreased during treatment. Compared with straight adults, bisexual adults and adults reporting additional sexual orientation groups exhibited statistically significantly less steep initial reductions in anxiety and depression symptoms. Compared with female adults, male adults had statistically significantly steeper initial reductions in anxiety and depression symptoms. Across these outcomes, statistically significant differences by gender identity and sexual orientation groups were small.

**Discussion:**

This employer-offered telemental health program provided clinically beneficial services to populations with diverse gender identities and sexual orientations, suggesting a potential pathway for accessing equitable mental health care.

## Introduction 

Globally, depressive and anxiety disorders are common and leading contributors to disability.^1^ Adults with minoritized gender identities and sexual orientations are at even higher risk for anxiety and depression.^2–4^ The occurrence of these mental health conditions may be even greater among adults reporting both minoritized gender identities *and* sexual orientations.^3,5^ Applying minority stress theory, discrimination and stigma experienced by individuals with marginalized gender identities and sexual orientations likely result in unique stressors that may increase the risk of mental health conditions in these populations.^6,7^ In light of these disparities and contributing factors, it is essential to ensure that scalable mental health programs can effectively address the unique mental health concerns of adults with minoritized gender identities and sexual orientations.

Although there exists a strong body of research on the effectiveness of evidence-based psychological interventions for anxiety and depression,^8,9^ most randomized controlled trials (RCTs) have not reported data on sexual orientation and transgender and gender diverse (TGD) identities.^10^ This research gap extends to real-world evaluations of psychological interventions. Real-world evaluations are uniquely positioned to complement RCTs by assessing whether findings are generalizable to underrepresented groups,^11^ including those with minoritized gender identities and sexual orientations. The limited number of real-world studies that have investigated clinical outcomes of psychological interventions across either gender identity or sexual orientation have more often been conducted among participants receiving care in partial hospital, inpatient, or intensive outpatient settings;^12–14^ a population that generally includes individuals with more severe symptoms. One exception, the large-scale evaluation of the United Kingdom’s (UK) Talking Therapies program, which provides both high-intensity and low-intensity outpatient therapy, found that bisexual participants and lesbian women were less likely to reliably recover from depression or anxiety relative to straight participants and straight women, respectively.^15^ Those disparate findings at scale highlight the importance of determining the types of evidence-based psychological interventions that are most effective across diverse populations.

Telemental health care, which includes telehealth-delivered psychotherapy, may be an approach to address potential mental health inequities across gender identity and sexual orientation. Telemental health programs could increase access to culturally competent care among marginalized populations that may not have support within their physical communities,^16–18^ including greater access to culturally competent care providers.^17,19^ They may also allow for the delivery of care with more privacy than some physical settings.^20^ However, similar to psychological interventions in general, there is a need for large-scale evaluations of these telemental health programs across gender identity and sexual orientation.

Therefore, the objective of this large-scale study was to evaluate anxiety and depression outcomes of an evidence-based, employer-offered telemental health program across individuals with different gender identities and sexual orientations in the United States, as well as among individuals with both minoritized gender identities *and* sexual orientations. As a secondary objective, this study also explored treatment satisfaction across gender identity and sexual orientation groups.

## Methods

### Design and participants

**FIG. 1.**
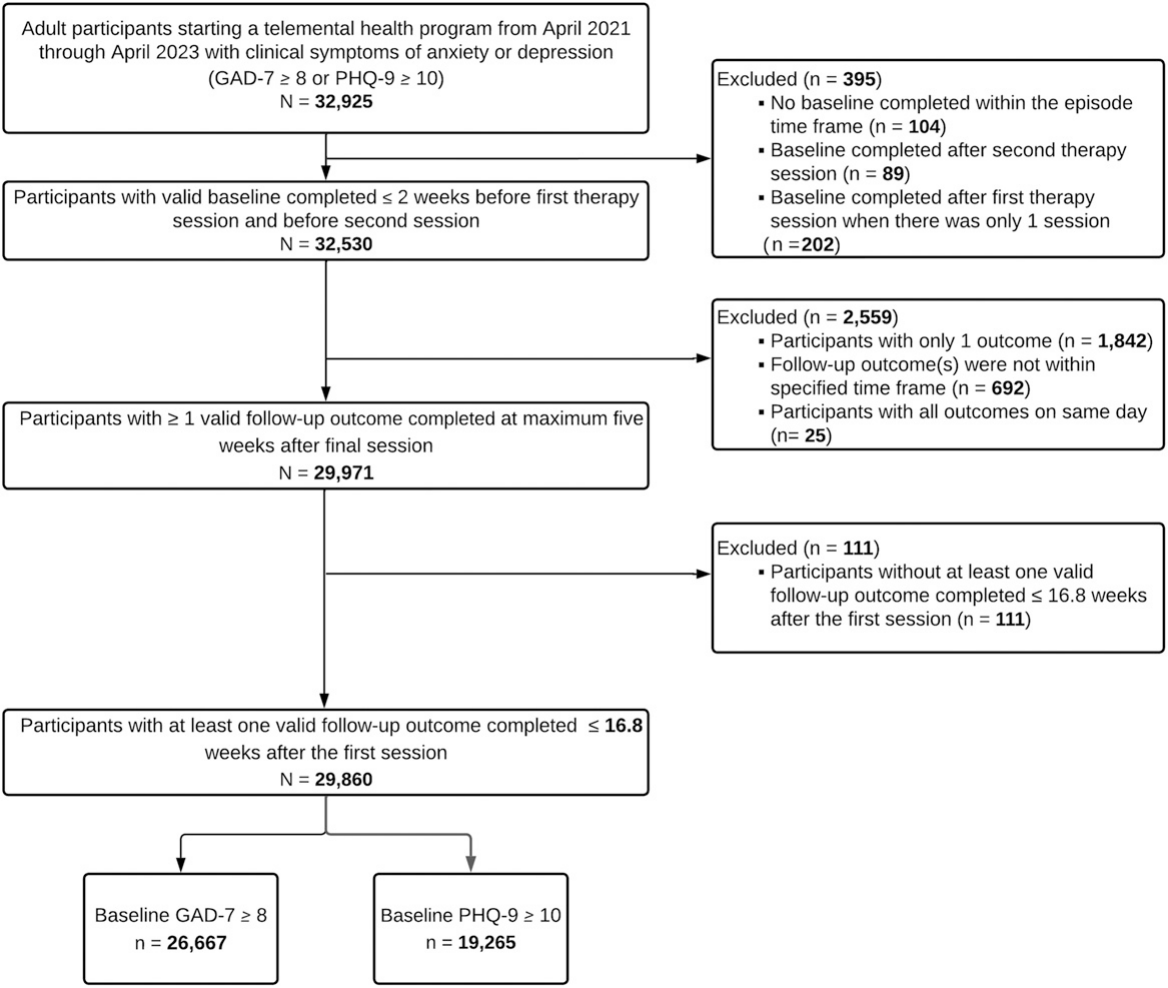
Participant study flow chart. GAD-7, 7-item Generalized Anxiety Disorder Scale; PHQ, 9-item Patient Health Questionnaire.

This study used a retrospective cohort of adults who received an evidence-based telemental health program between April 2021 and April 2023. The program was offered by Lyra Health and Lyra Clinical Associates to self-referring employees and dependents as an employer-sponsored benefit. Participants represented 50 states, the District of Columbia, U.S. territories, and freely associated states. Participants completed assessments including the Generalized Anxiety Disorder-7 (GAD-7) and Patient Health Questionnaire-9 (PHQ-9). Those with a baseline score in the clinical range on the GAD-7 (score ≥8) and/or PHQ-9 (score ≥10) were eligible for the study (*N* = 32,925).^21,22^ Participants were excluded if their completed assessments fell outside of the required timeframes (Fig. 1). The final sample included 29,860 participants (90.7% of those eligible), and the final dataset included 400,654 completed assessments (88.7% of eligible assessments; 200,327 GAD-7 and 200,327 PHQ-9). This analysis was determined exempt by the WCG Institutional Review Board (Princeton, NJ). All participants provided consent to take part in the telemental health program and, as a part of that consent, to have their de-identified data used for research purposes.

### Telemental health program design

Previous research provides a comprehensive description of the evidence-based telemental health program that was the target of this study.^23^ Briefly, the program merges synchronous video-based therapy sessions with asynchronous guided practice sessions, including digital activities, feedback, and messaging. All care components leverage evidence-based and transdiagnostic approaches.^24–26^ During the program onboarding process, participants have the option to search for therapists with expertise in the area in which they are seeking support (e.g., concerns around gender identity).

#### Culturally responsive care

The telemental health program incorporates culturally responsive care (CRC),^27–29^ grounding both evidence-based therapies and activities in fundamental CRC values of cultural responsiveness, humility, and respect.^30^ Providers complete a webinar on CRC concepts, such as intersectionality, as well as power and privilege within the therapeutic relationship.^31,32^ Therapists have access to didactics and continuing education credits on working with clients identifying as lesbian, gay, bisexual, transgender, queer, or additional minoritized sexual orientation and gender identities (LGBTQ+), as well as individual and group consultation with CRC specialists. The program also incorporates internal assessments of quality assurance, including CRC therapeutic alliance.

CRC is also integrated into the development of digital activities (e.g., coping with and responding to LGBTQ+ microaggressions). Therapists assign specific digital activities to ensure they are aligned with clients’ unique case formulation and presenting concerns for therapy.^23^

### Study measures

#### Demographics

Participants self-reported demographic information at registration and intake, including age, race and ethnicity, gender identity, sexual orientation, and highest educational attainment. Supplementary Table S1 describes response options and re-categorizations due to small sample sizes.

For gender identity, participants were re-categorized as “Female,” “Male,” Transgender and Gender Diverse (“TGD”), or “Unknown.” For sexual orientation, participants were re-categorized as “Straight,” “Bisexual,” “Gay or Lesbian,” “Additional sexual orientation groups,” or “Unknown.” Supplementary Table S1 details gender identities that were re-classified as TGD, as well as sexual orientation groups that were re-classified as “additional sexual orientation groups.”

#### Anxiety and depression

The PHQ-9 and GAD-7 were sent weekly in care. Recovery was defined as a final assessment score falling below the clinical threshold (i.e., GAD-7 < 8, PHQ-9 < 10). Using previously defined reliable change indices,^33^ reliable improvement was defined as a reduction of ≥4 on the GAD-7 and ≥6 on the PHQ-9 by the final assessment.

#### Treatment satisfaction

Treatment satisfaction was optionally assessed after a participant’s final session, regardless of treatment completion status, by asking “How likely are you to recommend your Lyra therapist to someone with needs similar to yours?”. Responses were coded as: “Extremely unlikely” = 1; “Unlikely” = 2; “Neutral” = 3; “Likely” = 4; and “Extremely likely” = 5.

### Statistical analysis

Symptom-specific analyses were comprised of participants with clinical elevations on each measure (i.e., PHQ-9 ≥ 10 and/or GAD-7 ≥ 8). Gender identity and sexual orientation group differences in treatment satisfaction, care duration, and sessions attended were tested using Kruskal-Wallis non-parametric tests with Bonferroni-corrected Dunn’s *post hoc* comparisons.^34^ Differences in reliable improvement and/or recovery rates were examined using Chi-square independence tests. Differences between baseline and final scores (PHQ-9, GAD-7) were assessed using 2-tailed paired *t*-tests.

Growth curve analysis was performed to evaluate outcomes across gender identity and sexual orientation groups, with separate models for anxiety and depression. The primary rationale for growth curve analysis is its ability to model outcomes that are collected at non-uniform intervals both within and between participants.^35^ Growth curve modeling also allows for participant-level characteristics to be included as predictors of differences in treatment trajectories. Fixed linear and quadratic effects of time (month, month^2^) were included to account for rapid early improvements and gradual deceleration over treatment. Although prior work evaluating this telemental health program has expressed the time variable in weeks,^23,30^ to improve model convergence, we rescaled the time variable from weeks to months using a conversion factor of 4.35 weeks per month (see Supplementary Table S2 for detailed methods). Symptom reports were nested within participants, who were nested within providers. Random effects included intercepts for provider and participant levels in both models, as well as participant-level linear and quadratic random effects of time. Associations between sessions attended and clinical outcomes were tested via time-varying fixed effects of session count in the week preceding an outcome (previous 7 days) and the prior week (previous 8–14 days). To evaluate potential trajectory differences, interaction effects of gender identity and/or sexual orientation with the time-varying fixed effects were included (Reference groups: female [gender identity] and straight [sexual orientation]). Both models also included fixed effects of age and highest educational attainment, which are potential confounders described in prior research.^15^ Supplementary Tables S3 and S4 contain the point estimates and model fit statistics for our baseline and intermediate models, characterized by more limited sets of predictors. Model fit criteria (Akaike Information Criterion, Bayesian Information Criterion, Log Likelihood) support the selection of the final models described in our primary analysis.

Analyses were completed in Python v 3.11.6 and R v 4.3.1. For growth curve modeling, missing clinical outcome data were handled using the full-information restricted maximum likelihood estimator in the lme4 package, which provides unbiased estimates under the missing-at-random assumption.^36,37^

## Results

Table 1 includes participants’ demographic characteristics (*N* = 29,860). At baseline, 89.3% of participants reported clinically elevated anxiety symptoms (GAD-7 ≥ 8), and 64.5% reported clinically elevated depression symptoms (PHQ-9 ≥ 10).

**Table 1.** Baseline Participants’ Characteristics by Gender Identity and Sexual Orientation Groups

(a) By gender identity group									
Characteristic	Overall, *N* = 29,860	Female, *N* = 19,019	Male, *N* = 9,405	TGD, *N* = 415	Unknown, *N* = 1,021	Test statistic (F or χ^2^)	*p* value	Effect size	
Ethnicity, *n* (%)						1,084.467^a^	<0.001^a^	0.110^a^	
Asian or Pacific Islander	5,271 (17.7)	3,460 (18.2)	1,621 (17.2)	38 (9.16)	152 (14.9)				
Black or African American	2,428 (8.13)	1,663 (8.74)	656 (6.97)	37 (8.92)	72 (7.07)				
Hispanic or Latino	3,190 (10.7)	2,065 (10.9)	978 (10.4)	51 (12.3)	96 (9.42)				
Missing or undisclosed	871 (2.92)	434 (2.28)	233 (2.48)	9 (2.17)	195 (19.1)				
Multiple	2,426 (8.12)	1,605 (8.44)	705 (7.50)	60 (14.5)	56 (5.48)				
Other	603 (2.02)	344 (1.81)	229 (2.43)	6 (1.45)	24 (2.35)				
White	15,071 (50.5)	9,448 (49.7)	4,983 (53.0)	214 (51.6)	426 (41.7)				
Age						42.303^b^	<0.001^b^	0.004^b^	
Mean (SD)	33.31 (9.27)	33.10 (9.34)	33.68 (9.05)	29.94 (8.33)	35.30 (9.81)				
Education, *n* (%)						650.350^a^	<0.001^a^	0.104^a^	
College graduate or above	21,087 (70.6)	13,521 (71.1)	6,742 (71.7)	229 (55.2)	595 (58.3)				
Missing or unknown	831 (2.78)	455 (2.39)	217 (2.31)	6 (1.45)	153 (15.0)				
Not a college graduate	7,942 (26.6)	5,043 (26.5)	2,446 (26.0)	180 (43.4)	273 (26.7)				
Baseline GAD-7 ≥ 8, *n* (%)	26,667 (89.3)	17,066 (89.7)	8,305 (88.3)	370 (89.2)	926 (90.7)	15.562^a^	0.001^a^	0.023^a^	
Baseline GAD-7 ≥ 8, Mean (SD)	13.02 (3.69)	13.05 (3.68)	12.92 (3.68)	13.49 (3.95)	13.24 (3.82)	5.614^b^	<0.001^b^	0.001^b^	
Baseline PHQ-9 ≥ 10, *n* (%)	19,265 (64.5)	12,217 (64.2)	6,075 (64.6)	314 (75.7)	659 (64.5)	23.201^a^	<0.001^a^	0.028^a^	
Baseline PHQ-9 ≥ 10, Mean (SD)	14.64 (3.84)	14.68 (3.83)	14.47 (3.79)	16.00 (4.41)	14.80 (4.02)	18.003^b^	<0.001^b^	0.003^b^	

(b) By sexual orientation group									
Characteristic	Overall, *N* = 29,860	Bisexual, *N* = 1,643	Gay or lesbian, *N* = 1,227	Additional sexual orientation, *N* = 1,604	Straight, *N* = 23,290	Unknown, *N* = 2,096	Test statistic (F or χ^2^)	*p* value	Effect size
Ethnicity, *n* (%)							1,548.337^a^	<0.001^a^	0.114
Asian or Pacific Islander	5,271 (17.7)	189 (11.5)	131 (10.7)	227 (14.2)	4,312 (18.5)	412 (19.7)			
Black or African American	2,428 (8.13)	149 (9.07)	154 (12.6)	141 (8.79)	1,833 (7.87)	151 (7.20)			
Hispanic or Latino	3,190 (10.7)	212 (12.9)	195 (15.9)	162 (10.1)	2,394 (10.3)	227 (10.8)			
Missing or undisclosed	871 (2.92)	28 (1.70)	16 (1.30)	25 (1.56)	477 (2.05)	325 (15.5)			
Multiple	2,426 (8.12)	199 (12.1)	106 (8.64)	191 (11.9)	1,791 (7.69)	139 (6.63)			
Other	603 (2.02)	25 (1.52)	19 (1.55)	32 (2.00)	482 (2.07)	45 (2.15)			
White	15,071 (50.5)	841 (51.2)	606 (49.4)	826 (51.5)	12,001 (51.5)	797 (38.0)			
Age							125.274^b^	<0.001^b^	0.017^b^
Mean (SD)	33.31 (9.27)	29.13 (7.91)	32.36 (8.70)	31.27 (8.74)	33.71 (9.28)	34.34 (9.77)			
Highest educational attainment, *n* (%)							1,032.739^a^	<0.001^a^	0.132^a^
College graduate or above	21,087 (70.6)	909 (55.3)	799 (65.1)	964 (60.1)	17,109 (73.5)	1,306 (62.3)			
Missing or unknown	831 (2.78)	34 (2.07)	31 (2.53)	23 (1.43)	505 (2.17)	238 (11.4)			
Not a college graduate	7,942 (26.6)	700 (42.6)	397 (32.4)	617 (38.5)	5,676 (24.4)	552 (26.3)			
Baseline GAD-7 ≥ 8, *n* (%)	26,667 (89.3)	1,470 (89.5)	1,085 (88.4)	1,395 (87.0)	20,815 (89.4)	1,902 (90.7)	14.854^a^	0.005^a^	0.022^a^
Baseline GAD-7 ≥ 8, Mean (SD)	13.02 (3.69)	13.26 (3.65)	13.21 (3.84)	13.12 (3.78)	12.97 (3.66)	13.19 (3.80)	4.549^b^	0.001^b^	0.001^b^
Baseline PHQ-9 ≥ 10, *n* (%)	19,265 (64.5)	1,242 (75.6)	832 (67.8)	1,148 (71.6)	14,689 (63.1)	1,354 (64.6)	150.031^a^	<0.001^a^	0.071^a^
Baseline PHQ-9 ≥ 10, Mean (SD)	14.64 (3.84)	15.26 (4.07)	14.75 (3.91)	15.16 (4.05)	14.51 (3.77)	14.99 (4.04)	20.422^b^	<0.001^b^	0.004^b^

Column percentages sum to 100.

^a^ Chi-square test.

^b^ ANOVA test.

GAD-7, 7-item Generalized Anxiety Disorder Scale; PHQ, 9-item Patient Health Questionnaire; SD, standard deviation; TGD, Transgender and gender diverse.

### Paired samples *t*-tests and effect sizes

Participants with a baseline GAD-7 ≥ 8 reported an average final GAD-7 score of 6.19 (standard deviation [SD] = 4.48), and those with a baseline PHQ-9 ≥ 10 reported an average final PHQ-9 score of 6.77 (SD = 5.18). The average decline in GAD-7 score was significant (*M*_Difference_ = 6.83, SD = 5.01; *t*(26666) = 222.6, *p* < 0.001; Hedges’ *g* = 1.66), as was the average decline in PHQ-9 score (*M*_Difference_ = 7.87, SD = 5.50; *t*(19264) = 198.48, *p* < 0.001; Hedges’ *g* = 1.71).

### Reliable improvement or recovery

Statistically significant differences in rates of reliable improvement and/or recovery in either anxiety or depression symptoms were detected across gender identity and sexual orientation groups (Table 2). Effect sizes were small (Cramer’s *V* ranges: 0.019–0.039). Rates of reliable improvement or recovery in either anxiety or depression symptoms ranged from 82.4% to 87.5% across gender identity groups (*χ*^2^(3)* =* 19.52, *p* < 0.001, Cramers’ *V* = 0.026), and from 84.3% to 86.9% across sexual orientation groups (*χ*^2^(4)* =* 18.39, *p* = 0.001, Cramers’ *V* = 0.025).

**Table 2.** Rates of Reliable Improvement and/or Recovery in Symptoms of Anxiety (GAD-7) or Depression (PHQ-9) Across Gender Identity and Sexual Orientation Groups

(a) By gender identity group									
Characteristic	Overall, *N* = 29,860	Female, *N* = 19,019	Male, *N* = 9,405	TGD, *N* = 415	Unknown, *N* = 1,021	χ^2^ test statistic	*p* value	χ^2^ effect size	
Reliable improvement, *n* (%)^a^	23,616 (79.1)	14,982 (78.8)	7,530 (80.1)	316 (76.1)	788 (77.2)	10.973	0.012	0.019	
Recovery, *n* (%)^b^	23,414 (78.4)	14,771 (77.7)	7,559 (80.4)	297 (71.6)	787 (77.1)	40.184	<0.001	0.037	
Reliable improvement and recovery*, n* (%)^c^	20,852 (69.8)	13,135 (69.1)	6,753 (71.8)	266 (64.1)	698 (68.4)	30.200	<0.001	0.032	
Reliable improvement or recovery, *n* (%)^d^	25,818 (86.5)	16,381 (86.1)	8,231 (87.5)	342 (82.4)	864 (84.6)	19.516	<0.001	0.026	

**(b) By sexual orientation group**									
**Characteristic**	**Overall, *N* = 29,860**	**Bisexual,** ***N* = 1,643**	**Gay or lesbian, *N* = 1,227**	**Additional sexual orientation,** ***N* = 1,604**	**Straight, *N* = 23,290**	**Unknown, *N* = 2,096**	**χ^2^ test statistic**	***p* value**	**χ^2^ effect size**
Reliable improvement, *n* (%)^a^	23,616 (79.1)	1,266 (77.1)	968 (78.9)	1,225 (76.4)	18,539 (79.6)	1,618 (77.2)	19.539	<0.001	0.026
Recovery, *n* (%)^b^	23,414 (78.4)	1,213 (73.8)	946 (77.1)	1,211 (75.5)	18,443 (79.2)	1,601 (76.4)	43.076	<0.001	0.038
Reliable improvement and recovery, *n* (%)^c^	20,852 (69.8)	1,070 (65.1)	835 (68.1)	1,067 (66.5)	16,478 (70.8)	1,402 (66.9)	45.433	<0.001	0.039
Reliable improvement or recovery, *n* (%)^d^	25,818 (86.5)	1,387 (84.4)	1,062 (86.6)	1,352 (84.3)	20,231 (86.9)	1,786 (85.2)	18.388	0.001	0.025

^a^ Reliable improvement: ≥ 4 point decrease on the final GAD-7 among those with baseline GAD-7 ≥ 8, and/or ≥6 point decrease on the final PHQ-9 among those with baseline PHQ-9 ≥ 10.

^b^ Recovery: Final GAD-7 < 8 among those with baseline GAD-7 ≥ 8, and/or final PHQ-9 < 10 among those with baseline PHQ-9 ≥ 10.

^c^ Reliable Improvement and Recovery: ≥ 4 point decrease on the final GAD-7 and final GAD-7 < 8 among those with baseline GAD-7 ≥ 8, and/or ≥6 point decrease on the final PHQ-9 and final PHQ-9 < 10 among those with baseline PHQ-9 ≥ 10.

^d^ Reliable Improvement or Recovery: ≥ 4 point decrease on the final GAD-7 or final GAD-7 < 8 among those with baseline GAD-7 ≥ 8, and/or ≥6 point decrease on the final PHQ-9 or final PHQ-9 < 10 among those with baseline PHQ-9 ≥ 10.

Post hoc tests revealed a higher rate of reliable improvement or recovery among male participants (87.5%), compared to participants reporting all other gender identities (Range: 82.4 − 86.1%; Residual = 3.61, *p* = 0.002). The rate of reliable improvement or recovery was also higher among straight participants (86.9%) relative to participants reporting all other sexual orientation groups (Range: 84.3 − 86.6%; Residual = 3.82, *p* = 0.001). Additional reliable improvement or recovery metrics are available in Table 2 and Supplementary Table S5.

### Gender and sexual orientation differences in trajectories of anxiety and depression symptoms

Models included fixed coefficients of age and highest educational attainment (Table 3).

**Table 3.** Growth Curve Modeling Results of Anxiety and Depression Across Gender Identity and Sexual Orientation Group, *b* (95% Confidence Interval)

	Anxiety symptoms (GAD-7)^a^	Depression symptoms (PHQ-9)^b^
Intercept	11.78 (11.71, 11.85)^***^	12.93 (12.84, 13.02)^***^
Month	−4.52 (−4.61, −4.44)^***^	−5.35 (−5.46, −5.23)^***^
Month^2^	0.86 (0.84, 0.89)^***^	1.03 (0.99, 1.07)^***^
Gender		
Male	−0.13 (−0.24, −0.03)^*^	−0.06 (−0.19, 0.08)
TGD	−1.13 (−2.33, 0.07)	−0.59 (−2.08, 0.90)
Sexual orientation		
Bisexual	0.20 (−0.01, 0.41)	0.52 (0.26, 0.77)^***^
Gay or lesbian	0.40 (0.04, 0.75)^*^	0.49 (0.05, 0.93)^*^
Additional sexual orientation	−0.04 (−0.28, 0.20)	0.40 (0.11, 0.69)^**^
Session last 7 days	−0.93 (−0.97, −0.88)^***^	−0.99 (−1.05, −0.94)^***^
Session last 8–14 days	−0.71 (−0.75, −0.66)^***^	−0.81 (−0.86, −0.75)^***^
Highest educational attainment		
Missing or unknown	0.39 (0.13, 0.66)^**^	0.39 (0.03, 0.75)^*^
Not a college graduate	0.53 (0.44, 0.62)^***^	0.97 (0.86, 1.08)^***^
Age (in years, mean-centered)	−0.003 (−0.01, 0.001)	−0.01 (−0.01, 0.0001)
Gender × sexual orientation		
Male × bisexual	0.29 (−0.34, 0.93)	−0.03 (−0.80, 0.73)
TGD × bisexual	1.12 (−0.43, 2.68)	1.06 (−0.82, 2.93)
Male × gay or lesbian	−0.21 (−0.67, 0.25)	−0.54 (−1.12, 0.04)
TGD × gay or lesbian	1.94 (0.27, 3.61)^*^	0.58 (−1.50, 2.65)
Male × additional sexual orientation	0.08 (−0.46, 0.62)	−0.49 (−1.14, 0.16)
TGD × additional sexual orientation	1.57 (0.26, 2.88)^*^	2.00 (0.39, 3.62)^*^
Month × gender		
Month × male	−0.32 (−0.47, −0.17)^***^	−0.26 (−0.46, −0.06)^**^
Month × TGD	1.36 (−0.52, 3.24)	0.76 (−1.48, 2.99)
Month^2^ × gender		
Month^2^ × male	0.05 (0.003, 0.10)^*^	0.01 (−0.05, 0.07)
Month^2^ × TGD	−0.41 (−1.05, 0.24)	−0.09 (−0.87, 0.69)
Month × sexual orientation		
Month × bisexual	0.43 (0.14, 0.73)^**^	0.72 (0.36, 1.08)^***^
Month × gay or lesbian	0.06 (−0.46, 0.57)	−0.06 (−0.71, 0.58)
Month × additional sexual orientation	0.37 (0.03, 0.71)^*^	0.66 (0.24, 1.07)^**^
Month^2^ × sexual orientation		
Month^2^ × bisexual	−0.12 (−0.21, −0.02)^*^	−0.23 (−0.34, −0.11)^***^
Month^2^ × gay or lesbian	−0.05 (−0.22, 0.11)	−0.03 (−0.24, 0.18)
Month^2^ × additional sexual orientation	−0.11 (−0.21, −0.001)^*^	−0.20 (−0.33, −0.07)^**^
Session last 7 days × gender		
Session last 7 days × male	0.08 (0.01, 0.15)^*^	0.01 (−0.08, 0.10)
Session last 7 days × TGD	0.28 (−0.63, 1.19)	0.06 (−1.07, 1.19)
Session last 7 days × sexual orientation		
Session last 7 days × bisexual	−0.04 (−0.18, 0.10)	−0.20 (−0.37, −0.03)^*^
Session last 7 days × gay or lesbian	−0.05 (−0.30, 0.20)	0.01 (−0.30, 0.32)
Session last 7 days × additional sexual orientation	0.20 (0.04, 0.36)^*^	0.09 (−0.11, 0.28)
Session last 8–14 days × gender		
Session last 8–14 days × male	0.01 (−0.07, 0.08)	0.01 (−0.08, 0.11)
Session last 8–14 days × TGD	0.30 (−0.68, 1.29)	0.16 (−1.01, 1.33)
Session last 8–14 days × sexual orientation		
Session last 8–14 days × bisexual	0.14 (−0.01, 0.28)	0.04 (−0.13, 0.21)
Session last 8–14 days × lesbian or gay	0.07 (−0.18, 0.33)	0.01 (−0.31, 0.32)
Session last 8–14 days × additional sexual orientation	0.21 (0.04, 0.37)^*^	0.21 (0.01, 0.41)^*^
Month × gender × sexual orientation		
Month × male × bisexual	–0.57 (−1.51, 0.36)	−0.17 (−1.33, 0.99)
Month × TGD × bisexual	−0.90 (−3.23, 1.43)	−0.88 (−3.63, 1.87)
Month × male × gay or lesbian	0.35 (−0.32, 1.02)	0.54 (−0.31, 1.39)
Month × TGD × gay or lesbian	−2.00 (−4.45, 0.45)	−1.39 (−4.40, 1.62)
Month × male × additional sexual orientation	0.15 (−0.64, 0.94)	−0.15 (−1.09, 0.79)
Month × TGD × additional sexual orientation	−1.96 (−3.98, 0.06)	−1.06 (−3.47, 1.35)
Month^2^ × gender × sexual orientation		
Month^2^ × male × bisexual	0.16 (−0.14, 0.46)	0.05 (−0.31, 0.42)
Month^2^ × TGD × bisexual	0.43 (−0.34, 1.21)	0.27 (−0.66, 1.21)
Month^2^ × male × gay or lesbian	−0.03 (−0.25, 0.18)	−0.08 (−0.35, 0.19)
Month^2^ × TGD × gay or lesbian	0.53 (−0.28, 1.35)	0.32 (−0.68, 1.32)
Month^2^ × male × additional sexual orientation	0.03 (−0.23, 0.28)	0.11 (−0.19, 0.41)
Month^2^ × TGD × additional sexual orientation	0.64 (−0.04, 1.33)	0.19 (−0.64, 1.02)
Session last 7 days × gender × sexual orientation		
Session last 7 days × male × bisexual	0.01 (−0.42, 0.44)	0.12 (−0.39, 0.63)
Session last 7 days × TGD × bisexual	−0.82 (−1.93, 0.29)	−0.13 (−1.47, 1.21)
Session last 7 days × Male × gay or lesbian	0.03 (−0.30, 0.36)	0.002 (−0.40, 0.40)
Session last 7 days × TGD × gay or lesbian	−0.63 (−1.84, 0.58)	0.10 (−1.41, 1.60)
Session last 7 days × Male × additional sexual orientation	−0.34 (−0.71, 0.04)	−0.05 (−0.48, 0.37)
Session last 7 days × TGD × additional sexual orientation	−0.42 (−1.40, 0.56)	−0.16 (−1.37, 1.05)
Gender × sexual orientation × session lasts 8–14 days		
Session last 8–14 days × male × bisexual	−0.04 (−0.49, 0.42)	0.09 (−0.46, 0.64)
Session last 8–14 days × TGD × bisexual	−0.71 (−1.90, 0.47)	−0.62 (−2.00, 0.75)
Male × gay or lesbian × session last 8–14 days	−0.17 (−0.50, 0.17)	−0.01 (−0.42, 0.40)
TGD × gay or lesbian × session last 8–14 days	−0.37 (−1.63, 0.90)	0.82 (−0.71, 2.35)
Male × additional sexual orientation × session last 8–14 days	0.21 (−0.16, 0.58)	−0.02 (−0.44, 0.41)
TGD × additional sexual orientation × session last 8–14 days	−0.49 (−1.54, 0.57)	−0.39 (−1.64, 0.85)

Reference category: straight, female, college graduate or above.

^a^ Analysis only included participants with baseline GAD-7 ≥ 8.

^b^ Analysis only included participants with baseline PHQ-9 ≥ 10.

^*^ *p* < 0.05; ^**^p < 0.01; ^***^p < 0.001.

#### Anxiety symptoms

The month coefficient (*b* = −4.52 [−4.61, −4.44]) indicates that GAD-7 scores declined by 4.52 points per month (1.04-point reduction per week) during the initial phase of care, and the month^2^ coefficient (*b* = 0.86 [0.84, 0.89]) suggests the rate of anxiety symptom improvement attenuated over time for the reference group.

Male participants, bisexual participants, and participants reporting additional sexual orientation groups experienced significantly different changes in anxiety symptoms over time relative to the reference groups. More specifically, relative to female participants, male participants experienced stronger initial declines in anxiety symptoms (month × male *b* = −0.32 [−0.47, −0.17] or 0.07 points faster per week) and symptoms improvements plateaued faster (month^2^ × male *b* = 0.05 [0.003, 0.10]). Relative to straight participants, participants identifying as bisexual and additional sexual orientation groups exhibited slower initial improvements in anxiety symptoms over time (month × bisexual *b* = 0.43 [0.14, 0.73] or 0.1 points per week; additional sexual orientation groups *b* = 0.37 [0.03, 0.71] or 0.08 points per week). These groups also exhibited a slower plateau of anxiety symptoms (month^2^ × bisexual *b* = −0.12 [−0.21, −0.02]; month^2^ × additional sexual orientation groups *b* = −0.11 [−0.21, −0.001]). No 3-way interactions involving both gender identity and sexual orientation groups were statistically significant (e.g., month × female × bisexual). Meaning, participants identifying as both minoritized gender identities and sexual orientations did not experience differences in anxiety outcomes, relative to the reference group.

Completion of each therapy session over the last 7 days was associated with a reduction in anxiety symptoms (*b* = −0.93 [−0.97, −0.88]). Relative to the reference groups, this reduction was less pronounced among male participants (*b* = 0.08 [0.01, 0.15]) and those identifying as additional sexual orientations (*b* = 0.20 [0.04, 0.36]). Each session completed in the last 8–14 days was also associated with a reduction in anxiety symptoms (*b* = −0.71 [−0.75, −0.66]), which was less pronounced among participants reporting additional sexual orientation groups (*b* = 0.21 [0.04, 0.37]) relative to the reference group.

#### Depression symptoms

The pattern of findings for the PHQ-9 models was very similar to those observed for the GAD-7 analysis (Table 3). However, the month^2^ × male interaction failed to reach significance, suggesting depression symptom improvements did not plateau at different rates. Additionally, the 2-way interactions involving sessions during the past 7 days (i.e., sessions × male, sessions × additional sexual orientation) observed in the anxiety analysis failed to reach significance, suggesting the effects of past-week sessions on depression symptoms did not differ among male participants or those reporting additional sexual orientation groups relativeto the reference groups. Finally, a significant session during the past 7 days × bisexual interaction was observed (*b* = −0.20 [−0.37, −0.03]), suggesting bisexual participants exhibited a larger decline in depression symptoms for each past-week session completed relative to straight participants. The 3-way interactions of time, gender identity, and sexual orientation groups were not statistically significant. Participants reporting both minoritized gender identities and sexual orientations did not experience differential depression outcomes compared to the reference group.

### Treatment Duration and Satisfaction

Table 4 reports median treatment duration and session completion by gender identity and sexual orientation groups (see Supplementary Table S6 for post hoc tests). Participants completed a median of 6 (interquartile range [IQR]: 4–8) sessions over 8.15 weeks (IQR: 4.03–12.74).

**Table 4.** Treatment Duration and Satisfaction Scores Across Gender Identity and Sexual Orientation Groups

(a) By gender identity group								
Characteristic	Overall, *N* = 29,860	Female, *N* = 19,019	Male, *N* = 9,405	TGD, *N* = 415	Unknown, *N* = 1,021	H statistic^a^	*p* value	Eta^2^ effect size^a^
Number of sessions completed, Median (IQR)	6 (4–8)	6 (4–8)	6 (4–8)	6 (4–8)	6 (3–8)	11.113	0.011	0.000^c^
Duration of care (weeks), Median (IQR)	8.15 (4.03–12.74)	8.28 (4.10–12.86)	8.03 (4.13–12.53)	8.17 (4.00–12.88)	7.43 (3.38–12.03)	16.804	<0.001	0.001

**Treatment Satisfaction**								
**Satisfaction reported, *N* (%)**	**Overall, *N* = 8,195** **(27%)**	**Female, *N* = 5,301** **(28%)**	**Male, *N* = 2,494** **(27%)**	**TGD, *N* = 126** **(30%)**	**Unknown, *N* = 274** **(27%)**	**H statistic^a^**	***p* Value**	**Eta^2^ effect size^a^**
Treatment Satisfaction Score^b^						30.370	<0.001	0.004
Mean (SD)	4.53 (0.78)	4.56 (0.76)	4.48 (0.80)	4.40 (0.81)	4.43 (0.89)			
Median (IQR)	5.00 (4.00, 5.00)	5.00 (4.00, 5.00)	5.00 (4.00, 5.00)	5.00 (4.00, 5.00)	5.00 (4.00, 5.00)			

(b) By sexual orientation group								
	**Overall**, *N* = 29,860	**Bisexual**, *N* = 1,643	**Gay or lesbian**, *N* = 1,227	**Additional sexual orientation**, *N* = 1,604	**Straight**, *N* = 23,290	**Unknown**, *N* = 2,096	**H statistic^a^**	***p* value**	**Eta^2^ effect size^a^**
Number of sessions completed, Median (IQR)	6 (4–8)	6 (4–8)	6 (4–8)	6 (4–8)	6 (4–8)	6 (4–8)	13.921	0.008	0.000
Duration of care (weeks), Median (IQR)	8.15 (4.03–12.74)	8.17 (4.00–12.79)	8.01 (4.00–12.43)	8.15 (4.01–12.96)	8.16 (4.14–12.74)	7.86 (3.51–12.71)	12.427	0.014	0.000

Treatment Satisfaction								
**Satisfaction Reported, *N* (%)**	**Overall, *N* = 8,195 (27%)**	**Bisexual, *N* = 449 (27%)**	**Gay or lesbian, *N* = 318 (26%)**	**Additional sexual orientation, *N* = 452 (28%)**	**Straight, *N* = 6,399 (27%)**	**Unknown, *N* = 577 (28%)**	**H statistic^a^**	***p* Value**	**Eta^2^ effect size^a^**
Treatment Satisfaction Score^b^							11.128	0.025	0.001
Mean (SD)	4.53 (0.78)	4.53 (0.84)	4.51 (0.85)	4.55 (0.72)	4.53 (0.77)	4.43 (0.85)			
Median (IQR)	5.00 (4.00, 5.00)	5.00 (4.00, 5.00)	5.00 (4.00, 5.00)	5.00 (4.00, 5.00)	5.00 (4.00, 5.00)	5.00 (4.00, 5.00)			

^a^ Kruskal–Wallis test.

^b^ Participants were asked, “*How likely are you to recommend your Lyra therapist to someone with needs similar to yours?”*

^c^ Kruskal–Wallis rank tests may yield a statistically significant result even when the median and IQR are identical across groups, in the case where one group stochastically dominates another. In this case, the near-zero effect size indicates that the magnitude of the difference was very small, though statistically significant.

Overall, 27.4% (8,195/29,860) of participants self-reported treatment satisfaction (Table 4), as such, these results were exploratory in nature. There were no significant differences in response rates across gender identity (*χ*^2^(3)* =* 7.77, *p* = 0.05) or sexual orientation (*χ*^2^(4)* =* 1.90, *p* = 0.75) groups. The average score was 4.53 (SD = 0.78). Across gender identity groups, the average score ranged from 4.40 (SD = 0.81) to 4.56 (SD = 0.76). There were small differences in treatment satisfaction across gender identity groups (H = 30.37, *p* < 0.001, Eta^2^ = 0.004), with female participants having higher scores than male or TGD participants (Supplementary Table S6). Across sexual orientation, average scores ranged from 4.43 (SD = 0.85) to 4.55 (SD = 0.72), and differences were observed (H = 11.13, *p* = 0.025, Eta^2^ = 0.001). Post hoc tests revealed lower satisfaction among participants with unknown sexual orientations than bisexual and straight participants (Supplementary Table S6).

## Discussion

These results provide some of the first evidence that an evidence-based telemental health program can be beneficial for the treatment of clinically elevated anxiety and depression symptoms across gender identity and sexual orientation in a U.S.-based population, including among participants reporting minoritized identities. Although participants reporting minoritized gender identities and sexual orientations experienced statistically significantly lower rates of improvement, rates of reliable improvement or recovery in anxiety or depression symptoms were high across all groups (≥82%). Additionally, anxiety and depression symptoms significantly declined over the course of treatment, with large effect sizes. Completion of therapy sessions during the past week and the past 8–14 days was also associated with improvements in symptoms.

As the first U.S.-based large-scale evaluation of a mental health program examining outcomes across both gender identity and sexual orientation, as well as their intersection, these data make significant contributions to the understanding of treating anxiety and depression across populations with diverse identities. In contrast, a large-scale evaluation of the UK’s Talking Therapies program reported clinical outcomes across sexual orientation groups by gender identity,^15^ but not minoritized gender identities. Differences in outcomes across sexual orientation were observed in both US and UK studies. In growth curve analysis, this study found slightly less pronounced symptom reductions for participants in the bisexual and additional sexual orientation groups. Bisexual individuals, in particular, experience distinct mental health challenges, such as less affirmation and support for their identity,^38,39^ suggesting that they may have unique needs requiring additional research.

Additional differential outcomes were observed across gender identity and sexual orientation in growth curve models. For example, female participants experienced less pronounced initial symptom declines compared to male individuals. The effects of each completed therapy session over the past week and past 8–14 weeks on clinical symptoms also varied across some gender identity and sexual orientation groups. Notably, all observed statistically significant differences were small and are not likely to be clinically significant. In addition, the current study did not find significant differences in trajectories of clinical symptom reductions among those with both minoritized gender and sexual identities. This population may be even more disproportionately affected by mental health disparities for numerous reasons,^3,5^ such as a higher likelihood of experiencing more frequent and severe discrimination due to possessing multiple minoritized identities.^5,6^ Yet, other research has not found individuals with both minoritized gender and sexual identities to have worse mental health outcomes than those reporting one minoritized identity.^40^ This indicates that some individuals with intersecting minoritized identities may develop greater resilience to cope with experiences of discrimination.^40–42^ Ongoing monitoring of mental health programs to ensure they meet the needs of marginalized individuals, including those with intersecting minoritized identities, is essential, as the current research in this area is limited. Additionally, discerning the cause of observed differences in this study could inform approaches addressing the unique mental health needs of certain gender identity and sexual orientation groups.

Prior research has typically reported poorer health care experiences among adults with minoritized gender identities and sexual orientations.^43,44^ In this study, average treatment satisfaction was high. However, small, albeit statistically significant, differences were observed across gender identity and sexual orientation groups. Given the exploratory nature of the satisfaction results due to low response rates, future research should assess factors that may impact treatment reporting and satisfaction levels in these populations.

A unique quality of the evidence-based telemental health program evaluated in this study was its programmatic inclusion of CRC. For example, this program provided tailored support for stressors experienced by LGBTQ+ participants. Treatment also incorporated a robust system of provider support, with clinical training, clinical consultations, and quality checks that incorporated CRC approaches. While these factors may help explain the program’s positive findings, more evidence is still needed, including more research on the effectiveness of specific culturally responsive clinical training programs and their components.^45^ Additional opportunities for future research include evaluating treatment fidelity across gender identity and sexual orientation.

### Strengths and limitations

Strengths of this study include a large sample to evaluate the novel intersection of gender identity and sexual orientation in the US. The real-world design highlights the generalizability of the findings to everyday clinical practice. However, although there was racial and ethnic diversity, the sample largely comprised employed adults or dependents with high levels of education. In addition, while the PHQ-9 and GAD-7 are well-validated measures,^21,22^ scores may be inflated for adults self-reporting minoritized gender identities and sexual orientations.^46^ More research is needed in this area, particularly across additional sexual orientations and gender identity groups. Despite the large sample, several gender identities and sexual orientations were conflated in the TGD and additional sexual orientation groups, respectively, due to small sub-group sample sizes. As a result, this study was unable to assess more specific differences in outcomes across these groups (e.g., participants identifying as intersex or pansexual), limiting conclusions for individuals with these identities. Future research should evaluate outcomes across more granular gender identity and sexual orientation groups to detect potential differences. Finally, because separate items were not used to assess participants’ sex assigned at birth and current gender identity, some participants’ gender identities may not have been fully captured. For example, in this study, a transgender participant who only selects female gender identity would be grouped with cisgender female participants.

### Health Equity Implications

As the first U.S.-based large-scale evaluation of its kind, this real-world study of an employer-offered, evidence-based telemental health program reported strong clinical outcomes and high treatment satisfaction across diverse gender identities and sexual orientations. The results of this study are complementary to findings of RCTs evaluating evidence-based psychological interventions for anxiety and depression, which have not commonly included data on TGD identities and sexual orientation,^10^ despite the evidence of mental health disparities.^2–4^ This study paves the way for future large-scale evaluations of evidence-based telemental health programs, including evaluating more granular outcomes across gender and sexual orientation identities and longer-term follow-up outcomes. Furthermore, community engagement for equity-oriented research remains critical to enhance trustworthiness and relevance. Additional opportunities for future research include LGBTQ+ community outreach and community-based input for this program. This research could inform future efforts to elucidate the potential role of employer-offered mental health benefits in promoting mental health among broader populations that include individuals with minoritized gender identities and sexual orientations.

## Abbreviations Used

CRC: Culturally responsive care
GAD-7: Generalized Anxiety Disorder-7
IQR: Interquartile range
LGBTQ+: Lesbian, gay, bisexual, transgender, queer, or additional minoritized sexual orientation and gender identities
PHQ-9: Patient Health Questionnaire-9
RCT: Randomized controlled trial
SD: Standard deviation
TGD: Transgender and gender diverse
UK: United Kingdom
