# Supplementary material for: Outcomes of an Evidence-Based Telemental Health Program Across Sexual Orientation and Gender Identity
Source: Health Equity. 2025 Oct 6;9(1):632–44. doi: 10.1177/24731242251382353 (PMC12670707; doi:10.1177/24731242251382353)
Supplement: Supplementary Tables [file 24731242251382353_supplementary_tables.docx]

**Table S1. Demographic Self-Identification Options: Additional Information**

| **Variable** | **Response Options** | **Re-Categorization** |
| --- | --- | --- |
| **Gender Identity Group^b^** | “Female” (Without Other Selections) | Female |
|  | “Male” (Without Other Selections) | Male |
|  | “Non-binary” | TGD^a^ |
|  | “Prefer not to say” | Unknown |
|  | “Genderqueer” | TGD^a^ |
|  | “Transgender” | TGD^a^ |
|  | “Not listed” | TGD^a^ |
|  | “Intersex” | TGD^a^ |
|  | “Two-spirit” | TGD^a^ |
|  | Missing | Unknown |
| **Sexual Orientation Group^c^** | “Straight” (Without Other Selections) | Straight |
|  | “Bisexual” (Without Other Selections) | Bisexual |
|  | “Gay” (Without Other Selections) | Gay or Lesbian |
|  | “Lesbian” (Without Other Selections) | Gay or Lesbian |
|  | “Pansexual” | Additional sexual orientation^a^ |
|  | “Asexual” | Additional sexual orientation^a^ |
|  | “Queer” | Additional sexual orientation^a^ |
|  | “Not listed” | Additional sexual orientation^a^ |
|  | “Prefer not to say” | Unknown |
|  | Missing | Unknown |
| **Race and Ethnicity^d^** | “American Indian or Alaska Native” | Other^a^ |
|  | “Asian or Pacific Islander” | Asian or Pacific Islander |
|  | “Black or African American” | Black or African American |
|  | “Hispanic or Latino” | Hispanic or Latino |
|  | “Native Hawaiian or Other Pacific Islander” | Other^a^ |
|  | “White” | White |
|  | “Prefer not to disclose” | Missing or undisclosed |
|  | “Other” | Other |
|  | Missing | Missing or undisclosed |
|  | Selected ≥2 race and ethnicity response options | Multiple |
| **Highest Educational Attainment^e^** |  | |
|  | “Less than 9th grade” | Not college graduate^a^ |
|  | “9th-11th grade (Includes 12th grade with no diploma)” | Not college graduate^a^ |
|  | “High school graduate/GED or equivalent” | Not college graduate^a^ |
|  | “Some college or AA degree” | Not college graduate^a^ |
|  | “College graduate or above” | College graduate or above |
|  | “Prefer Not to disclose” | Missing or unknown |
|  | Missing | Missing or unknown |

**Notes.** TGD: Transgender and gender diverse. ^a^Re-categorized due to small sample sizes. ^b^Participants were asked to select “Gender”; multiple options could be selected. ^c^Participants were asked to select “Sexual Orientation”; multiple options could be selected ^d^Participants were asked, “I identify as”; multiple options could be selected. ^e^Participants were asked, “What is your highest level of education completed?”

**Table S2.** Time Conversions Used for Growth Curve Modelling Results

| **Time units** | **Days** | **Weeks** | **Months** |
| --- | --- | --- | --- |
| **Formula** | N/A | Days ✕ (1➗7) | Days ✕ (12 ➗365) |
| **Conversions** | 1 | 0.14 | 0.03 |
|  | 7 | 1.00 | 0.23 |
|  | 14 | 2.00 | 0.46 |
|  | 21 | 3.00 | 0.69 |
|  | 28 | 4.00 | 0.92 |
| **1- Month Conversion Factor^a^** | 30.45 | 4.35 | 1.00 |
| ***Example*** | | | |
| **GAD-7 GCM Time Coefficient** |  | **Weeks (original)** | **Months (rescaled)** |
| Time |  | -1.04 (-1.06, -1.02)*** | -4.52 (-4.61, -4.44)*** |
| Time Squared |  | 0.05 (0.04, 0.05)*** | 0.86 (0.84, 0.89)*** |

**Notes**. GAD-7: 7-item Generalized Anxiety Disorder Scale; GCM: Growth curve model. ^a^This study used the 1-month conversion factor. Assuming no leap years, the 1-month conversion factor (4.35) was calculated using the following formula:  (365 days/12 months=30.417)/ 7 days.

**Table S3**. Growth Curve Modelling Results and Selection Metrics: Anxiety Symptoms (GAD-7), *b* (95% confidence interval)

| **Characteristics** | **Model 1** | | **Model 2** | | **Model 3** | | **Model 4** | | **Model 5** | | **Model 6 (Final Model)** | |
| --- | --- | --- | --- | --- | --- | --- | --- | --- | --- | --- | --- | --- |
| Month | -4.8 (-4.9, -4.8) *** | | -4.7 (-4.7, -4.6)*** | | -4.2 (-4.3, -4.1)*** | | -4.2(-4.3, -4.1)*** | | -4.2 (-4.3, -4.1)*** | | -4.5 (-4.6, -4.4)*** | |
| Month^2^ | 0.93 (0.91, 0.95)*** | | 0.87 (0.85, 0.89)*** | | 0.71 (0.69, 0.74)*** | | 0.71 (0.69, 0.74)*** | | 0.71 (0.69, 0.74)*** | | 0.86 (0.84, 0.89)*** | |
| Gender |  | | | | | | | | | | | |
| Male | -0.10 (-0.21, 0.00)* | | -0.13 (-0.23, -0.02)* | | -0.13 (-0.24, -0.03)* | | -0.14 (-0.25, -0.03)** | | -0.14 -0.25, -0.03)* | | -0.13 (-0.24, -0.03)* | |
| TGD | -0.81 (-2.0, 0.35) | | -0.92 (-2.1, 0.29) | | -1.1 (-2.3, 0.15) | | -1.1 (-2.4, 0.10) | | -1.1 (-2.4, 0.10) | | -1.1 (-2.3, 0.07) | |
| Sexual Orientation |  |  |  |  |  |  |  |  |  |  |  |  |
| Bisexual | 0.32 (0.12, 0.53)** | | 0.36 (0.15, 0.57)*** | | 0.33 (0.12, 0.55)** | | 0.23 (0.02, 0.45)* | | 0.21 (0.00, 0.43) | | 0.2 (-0.01, 0.41) | |
| Gay or Lesbian | 0.52 (0.17, 0.86)** | | 0.53 (0.17, 0.89)** | | 0.52 (0.16, 0.89)** | | 0.42 (0.05, 0.78)* | | 0.41 (0.04, 0.77)* | | 0.40 (0.04, 0.75)* | |
| Additional Sexual Orientation | 0.16 (-0.08, 0.39) | | 0.09 (-0.16, 0.33) | | 0.02 (-0.23, 0.27) | | -0.05 (-0.30, 0.19) | | -0.06 (-0.31, 0.18) | | -0.04 (-0.28, 0.20) | |
| Gender * Sexual Orientation |  |  |  |  |  |  |  |  |  |  |  |  |
| Male * Bisexual | 0.2 (-0.41, 0.82) | | 0.2 (-0.44, 0.84) | | 0.19 (-0.47, 0.84) | | 0.25 (-0.40, 0.90) | | 0.25 (-0.40, 0.90) | | 0.29 (-0.34, 0.93) | |
| TGD * Bisexual | 0.55 (-0.96, 2.1) | | 0.82 (-0.75, 2.4) | | 1.1 (-0.51, 2.7) | | 1.1 (-0.51, 2.7) | | 1.1 (-0.51, 2.7) | | 1.1 (-0.43, 2.7) | |
| Male * Gay or Lesbian | -0.31 (-0.76, 0.14) | | -0.34 (-0.81, 0.13) | | -0.33 (-0.80, 0.15) | | -0.22 (-0.70, 0.26) | | -0.22 (-0.69, 0.26) | | -0.21 (-0.67, 0.25) | |
| TGD * Gay or Lesbian | 1.3 (-0.26, 3.0) | | 1.6 (-0.06, 3.3) | | 1.8 (0.12, 3.6)* | | 1.9 (0.19, 3.6)* | | 1.9 (0.19, 3.6)* | | 1.9 (0.27, 3.6)* | |
| Male * Additional Sexual Orientation | -0.01 (-0.54, 0.51) | | 0.14 (-0.41, 0.69) | | 0.09 (-0.47, 0.65) | | 0.11 (-0.44, 0.67) | | 0.12 (-0.44, 0.68) | | 0.08 (-0.46, 0.62) | |
| TGD * Additional Sexual Orientation | 1.2 (-0.04, 2.5) | | 1.4 (0.06, 2.7)* | | 1.6 (0.24, 2.9)* | | 1.6 (0.27, 3.0)* | | 1.6 (0.27, 3.0)* | | 1.6 (0.26, 2.9)* | |
| Month * Gender |  | | | | | | | | | | | |
| Month * Male | -0.30 (-0.41, -0.19) *** | | -0.29 (-0.41, -0.18)*** | | -0.27 (-0.39, -0.15)*** | | -0.27 (-0.39, -0.15)*** | | -0.27 (-0.39, -0.15)*** | | -0.32 (-0.47, -0.17)*** | |
| Month * TGD | 1.4 (0.01, 2.8)* | | 1.4 (0.00, 2.9)* | | 1.1 (-0.47, 2.8) | | 1.2 (-0.44, 2.8) | | 1.2 (-0.44, 2.8) | | 1.4 (-0.52, 3.2) | |
| Month^2^ * Gender |  | | | | | | | | | | | |
| Month^2^ * Male | 0.04 (0.01, 0.08)* | | 0.04 (0.00, 0.08)* | | 0.03 (-0.01, 0.07) | | 0.03 (-0.01, 0.07) | | 0.03 (-0.01, 0.07) | | 0.05 (0.00, 0.10)* | |
| Month^2^ * TGD | -0.36 (-0.88, 0.16) | | -0.36 (-0.88, 0.17) | | -0.28 (-0.86, 0.31) | | -0.28 (-0.87, 0.30) | | -0.28 (-0.87, 0.30) | | -0.41 (-1.1, 0.24) | |
| Month * Sexual Orientation |  | | | | | | | | | | | |
| Month * Bisexual | 0.50 (0.28, 0.72)*** | | 0.53 (0.31, 0.75)*** | | 0.46 (0.22, 0.70)*** | | 0.46 (0.23, 0.70)*** | | 0.46 (0.23, 0.70)*** | | 0.43 (0.14, 0.73)** | |
| Month * Gay or Lesbian | 0.03 (-0.35, 0.41) | | 0.08 (-0.30, 0.46) | | 0.07 (-0.35, 0.49) | | 0.07 (-0.35, 0.49) | | 0.07 (-0.35, 0.49) | | 0.06 (-0.46, 0.57) | |
| Month * Additional Sexual Orientation | 0.56 (0.31, 0.80)*** | | 0.54 (0.29, 0.79)*** | | 0.41 (0.14, 0.68)** | | 0.41 (0.14, 0.68)** | | 0.41 (0.14, 0.68)** | | 0.37 (0.03, 0.71)* | |
| Month^2^ * Sexual Orientation |  |  |  |  |  |  |  |  |  |  |  |  |
| Month^2^ * Bisexual | -0.14 (-0.21, -0.07)*** | | -0.15 (-0.22, -0.08)*** | | -0.13 (-0.20, -0.05)** | | -0.13 (-0.20, -0.05)** | | -0.13 (-0.20, -0.05)** | | -0.12 (-0.21, -0.02)* | |
| Month^2^ * Gay or Lesbian | -0.03 (-0.16, 0.10) | | -0.06 (-0.19, 0.08) | | -0.05 (-0.19, 0.09) | | -0.05 (-0.19, 0.09) | | -0.05 (-0.19, 0.09) | | -0.05 (-0.22, 0.11) | |
| Month^2^ * Additional Sexual Orientation | -0.17 (-0.25, -0.09)*** | | -0.17 (-0.25, -0.09)*** | | -0.13 (-0.21, -0.04)** | | -0.13 (-0.21, -0.04)** | | -0.13 (-0.21, -0.04)** | | -0.11 (-0.21, 0.00)* | |
| Month * Gender * Sexual Orientation |  | | | | | | | | | | | |
| Month * Male * Bisexual | -0.49 (-1.2, 0.21) | | -0.5 (-1.2, 0.20) | | -0.53 (-1.3, 0.23) | | -0.54 (-1.3, 0.22) | | -0.54 (-1.3, 0.22) | | -0.57 (-1.5, 0.36) | |
| Month * TGD * Bisexual | -1.6 (-3.3, 0.16) | | -1.4 (-3.2, 0.36) | | -0.84 (-2.8, 1.1) | | -0.87 (-2.8, 1.1) | | -0.88 (-2.8, 1.1) | | -0.9 (-3.2, 1.4) | |
| Month * Male * Gay or Lesbian | 0.24 (-0.26, 0.74) | | 0.22 (-0.28, 0.72) | | 0.25 (-0.29, 0.80) | | 0.26 (-0.29, 0.80) | | 0.26 (-0.29, 0.80) | | 0.35 (-0.32, 1.0) | |
| Month * TGD * Gay or Lesbian | -1.7 (-3.6, 0.10) | | -2.0 (-3.8, -0.12)* | | -1.8 (-3.8, 0.24) | | -1.8 (-3.8, 0.22) | | -1.8 (-3.8, 0.22) | | -2 (-4.5, 0.45) | |
| Month * Male * Additional Sexual Orientation | 0.16 (-0.43, 0.74) | | 0.26 (-0.32, 0.85) | | 0.13 (-0.52, 0.77) | | 0.14 (-0.51, 0.78) | | 0.14 (-0.51, 0.78) | | 0.15 (-0.64, 0.94) | |
| Month * TGD * Additional Sexual Orientation | -2.3 (-3.8, -0.74)** | | -2.2 (-3.8, -0.70)** | | -1.9 (-3.6, -0.18)* | | -1.9 (-3.7, -0.21)* | | -1.9 (-3.7, -0.21)* | | -2 (-4.0, 0.06) | |
| Month^2^ * Gender * Sexual Orientation |  | | | | | | | | | | | |
| Month^2^ * Male * Bisexual | 0.13 (-0.10, 0.36) | | 0.14 (-0.09, 0.37) | | 0.14 (-0.12, 0.39) | | 0.14 (-0.12, 0.39) | | 0.14 (-0.12, 0.39) | | 0.16 (-0.14, 0.46) | |
| Month^2^ * TGD * Bisexual | 0.59 (-0.02, 1.2) | | 0.54 (-0.08, 1.2) | | 0.39 (-0.29, 1.1) | | 0.4 (-0.28, 1.1) | | 0.4 (-0.28, 1.1) | | 0.43 (-0.34, 1.2) | |
| Month^2^ * Male * Gay or Lesbian | -0.02 (-0.19, 0.15) | | 0 (-0.17, 0.17) | | -0.01 (-0.19, 0.17) | | -0.01 (-0.19, 0.17) | | -0.01 (-0.19, 0.17) | | -0.03 (-0.25, 0.18) | |
| Month^2^ * TGD * Gay or Lesbian | 0.38 (-0.26, 1.0) | | 0.43 (-0.21, 1.1) | | 0.4 (-0.30, 1.1) | | 0.4 (-0.30, 1.1) | | 0.4 (-0.30, 1.1) | | 0.53 (-0.28, 1.3) | |
| Month^2^ * Male * Additional Sexual Orientation | 0.02 (-0.17, 0.22) | | -0.02 (-0.22, 0.17) | | 0.02 (-0.20, 0.23) | | 0.02 (-0.20, 0.23) | | 0.02 (-0.20, 0.23) | | 0.03 (-0.23, 0.28) | |
| Month^2^ * TGD * Additional Sexual Orientation | 0.68 (0.13, 1.2)* | | 0.67 (0.11, 1.2)* | | 0.58 (-0.04, 1.2) | | 0.59 (-0.03, 1.2) | | 0.59 (-0.03, 1.2) | | 0.64 (-0.04, 1.3) | |
| Session Last 7 Days |  | | -0.81 (-0.85, -0.76)*** | | -0.93 (-0.97, -0.89)*** | | -0.93 (-0.98, -0.89)*** | | -0.93 (-0.98, -0.89)*** | | -0.93 (-0.97, -0.88)*** | |
| Gender * Session Last 7 Days |  |  |  |  |  |  |  |  |  |  |  |  |
| Male * Session Last 7 Days |  |  | 0.06 (-0.01, 0.14) | | 0.07 (0.00, 0.15) | | 0.07 (0.00, 0.15) | | 0.07 (0.00, 0.15) | | 0.08 (0.01, 0.15)* | |
| TGD * Session Last 7 Days |  |  | 0.23 (-0.68, 1.1) | | 0.3 (-0.62, 1.2) | | 0.27 (-0.66, 1.2) | | 0.27 (-0.66, 1.2) | | 0.28 (-0.63, 1.2) | |
| Sexual Orientation * Session Last 7 Days |  |  |  |  |  |  |  |  |  |  |  |  |
| Bisexual * Session Last 7 Days |  |  | -0.08 (-0.22, 0.07) | | -0.06 (-0.21, 0.08) | | -0.06 (-0.21, 0.08) | | -0.06 (-0.21, 0.08) | | -0.04 (-0.18, 0.10) | |
| Gay or Lesbian * Session Last 7 Days |  |  | -0.05 (-0.30, 0.21) | | -0.05 (-0.31, 0.21) | | -0.05 (-0.31, 0.21) | | -0.05 (-0.31, 0.21) | | -0.05 (-0.30, 0.20) | |
| Additional Sexual Orientation * Session Last 7 Days |  |  | 0.17 (0.01, 0.34)* | | 0.22 (0.05, 0.38)* | | 0.22 (0.05, 0.38)** | | 0.22 (0.05, 0.38)** | | 0.20 (0.04, 0.36)* | |
| Gender * Sexual Orientation * Session Last 7 Days |  |  |  |  |  |  |  |  |  |  |  |  |
| Male * Bisexual * Session Last 7 Days |  |  | 0.01 (-0.43, 0.45) | | 0.01 (-0.43, 0.46) | | 0.01 (-0.43, 0.46) | | 0.01 (-0.43, 0.46) | | 0.01 (-0.42, 0.44) | |
| TGD * Bisexual * Session Last 7 Days |  |  | -0.61 (-1.7, 0.51) | | -0.74 (-1.9, 0.39) | | -0.7 (-1.8, 0.43) | | -0.7 (-1.8, 0.43) | | -0.82 (-1.9, 0.29) | |
| Male * Gay or Lesbian * Session Last 7 Days |  |  | 0.04 (-0.29, 0.37) | | 0.04 (-0.30, 0.37) | | 0.04 (-0.29, 0.38) | | 0.04 (-0.29, 0.38) | | 0.03 (-0.30, 0.36) | |
| TGD * Gay or Lesbian * Session Last 7 Days |  |  | -0.5 (-1.7, 0.72) | | -0.58 (-1.8, 0.65) | | -0.56 (-1.8, 0.68) | | -0.56 (-1.8, 0.68) | | -0.63 (-1.8, 0.58) | |
| Male * Additional Sexual Orientation * Session Last 7 Days |  |  | -0.37 (-0.74, 0.01) | | -0.36 (-0.74, 0.02) | | -0.35 (-0.73, 0.02) | | -0.36 (-0.73, 0.02) | | -0.34 (-0.71, 0.04) | |
| TGD * Additional Sexual Orientation * Session Last 7 Days |  |  | -0.37 (-1.4, 0.62) | | -0.45 (-1.5, 0.55) | | -0.42 (-1.4, 0.58) | | -0.42 (-1.4, 0.58) | | -0.42 (-1.4, 0.56) | |
| Session Last 8-14 Days |  |  |  |  | -0.76 (-0.80, -0.71)*** | | -0.76 (-0.80, -0.71)*** | | -0.76 (-0.80, -0.71)*** | | -0.71 (-0.75, -0.66)*** | |
| Gender * Session Last 8-14 Days |  |  |  |  |  |  |  |  |  |  |  |  |
| Male * Session Last 8-14 Days |  |  |  |  | 0 (-0.07, 0.08) | | 0 (-0.07, 0.08) | | 0 (-0.07, 0.08) | | 0.01 (-0.07, 0.08) | |
| TGD * Session Last 8-14 Days |  |  |  |  | 0.48 (-0.52, 1.5) | | 0.46 (-0.55, 1.5) | | 0.46 (-0.55, 1.5) | | 0.3 (-0.68, 1.3) | |
| Sexual Orientation * Session Last 8-14 Days |  |  |  |  |  |  |  |  |  |  |  |  |
| Bisexual * Session Last 8-14 Days |  |  |  |  | 0.1 (-0.05, 0.25) | | 0.1 (-0.05, 0.24) | | 0.1 (-0.05, 0.24) | | 0.14 (-0.01, 0.28) | |
| Gay or Lesbian * Session Last 8-14 Days |  |  |  |  | 0.03 (-0.23, 0.29) | | 0.03 (-0.23, 0.29) | | 0.03 (-0.23, 0.29) | | 0.07 (-0.18, 0.33) | |
| Additional Sexual Orientation * Session Last 8-14 Days |  |  |  |  | 0.22 (0.06, 0.39)** | | 0.23 (0.06, 0.39)** | | 0.23 (0.06, 0.39)** | | 0.21 (0.04, 0.37)* | |
| Gender * Sexual Orientation * Session Last 8-14 Days |  | | | | | | | | | | | |
| Male * Bisexual * Session Last 8-14 Days |  |  |  |  | 0.04 (-0.42, 0.51) | | 0.04 (-0.42, 0.51) | | 0.04 (-0.42, 0.51) | | -0.04 (-0.49, 0.42) | |
| TGD * Bisexual * Session Last 8-14 Days |  |  |  |  | -0.9 (-2.1, 0.30) | | -0.88 (-2.1, 0.32) | | -0.88 (-2.1, 0.32) | | -0.71 (-1.9, 0.47) | |
| Male * Gay or Lesbian * Session Last 8-14 Days |  |  |  |  | -0.1 (-0.44, 0.23) | | -0.1 (-0.44, 0.24) | | -0.1 (-0.44, 0.24) | | -0.17 (-0.50, 0.17) | |
| TGD * Gay or Lesbian * Session Last 8-14 Days |  |  |  |  | -0.53 (-1.8, 0.76) | | -0.51 (-1.8, 0.78) | | -0.51 (-1.8, 0.78) | | -0.37 (-1.6, 0.90) | |
| Male * Additional Sexual Orientation * Session Last 8-14 Days |  |  |  |  | 0.2 (-0.18, 0.58) | | 0.2 (-0.18, 0.58) | | 0.2 (-0.18, 0.58) | | 0.21 (-0.16, 0.58) | |
| TGD * Additional Sexual Orientation * Session Last 8-14 Days |  |  |  |  | -0.64 (-1.7, 0.44) | | -0.61 (-1.7, 0.46) | | -0.61 (-1.7, 0.46) | | -0.49 (-1.5, 0.57) | |
| Educational Attainment |  |  |  |  |  |  |  |  |  |  |  |  |
| Missing or Unknown |  |  |  |  |  |  | 0.37 (0.09, 0.64)** | | 0.36 (0.09, 0.64)** | | 0.39 (0.13, 0.66)** | |
| Not college graduate |  |  |  |  |  |  | 0.53 (0.44, 0.62)*** | | 0.53 (0.44, 0.62)*** | | 0.53 (0.44, 0.62)*** | |
| Age (Years, Mean Centered) |  |  |  |  |  |  |  |  | 0.00 (-0.01, 0.00)* | | 0 (-0.01, 0.00) | |
| **Model Selection Metrics** | | | | | | | | | | | | |
| AIC | 900,490 | | 898,152 | | 896,180 | | 896,052 | | 896,050 | | 891,833 | |
| BIC | 900,901 | | 898,683 | | 896,831 | | 896,724 | | 896,732 | | 892,544 | |
| Log Likelihood | -450,204 | | -449,023 | | -448,025 | | -447,959 | | -447,957 | | -445,845 | |
| Chi-Square Difference Test (*P* Value) | -- | | 2,362 (<0.05) | | 1,996 (<0.05) | | 131 (<0.05) | | 4 (0.046) | | 4,224 (<0.05) | |

**Notes.** GAD-7: 7-item Generalized Anxiety Disorder Scale. Reference category: Straight, female, college graduate or above. Analysis only included participants with baseline GAD-7 ≥ 8.

^a^Model 1: Month, month^2^, gender identity, sexual orientation, gender identity * sexual orientation, month * gender identity,month^2^ * gender identity, month * sexual orientation, month^2^ * sexual orientation, month * gender identity * sexual orientation, month^2^* gender identity * sexual orientation, random effects for provider and patient nested under provider, random effect for month per patient nested under provider

^b^Model 2:  Model 1 + session last 7 days, session last 7 days * gender identity, session last 7 days * sexual orientation,  session last 7 days * gender identity * sexual orientation

^c^Model 3: Model 2 + session last 8-14 days, session last 8-14 days* gender identity,  session last 8-14 days * sexual orientation, session last 8-14 days * gender identity * sexual orientation

^d^Model 4: Model 3 + education

^e^Model 5: Model 4 + age (grand mean centered)

^f^Model 6: Model 5 + additional random effect term for month^2^ per clients nested under provider

*p<0.05; **p<0.01; ***p<0.001

**Table S4**. Growth Curve Modelling Results and Selection Metrics: Depression Symptoms (PHQ-9), *b* (95% confidence interval)

| **Characteristics** | **Model 1** | **Model 2** | **Model 3** | **Model 4** | **Model 5** | **Model 6 (Final Model)** |  |
| --- | --- | --- | --- | --- | --- | --- | --- |
| Month | -5.7 (-5.8, -5.6)*** | -5.5 (-5.6, -5.5)*** | -5.0 (-5.1, -4.9)*** | -5.0 (-5.1, -4.9)*** | -5.0 (-5.1, -4.9)*** | -5.3 (-5.5, -5.2)*** |  |
| Month^2^ | 1.1 (1.1, 1.1)*** | 1.0 (1.0, 1.1)*** | 0.86 (0.83, 0.89)*** | 0.86 (0.83, 0.89)*** | 0.86 (0.83, 0.89)*** | 1.0 (1.0, 1.1)*** |  |
| Gender |  | | | | | |  |
| Male | -0.05 (-0.19, 0.09) | -0.05 (-0.19, 0.10) | -0.06 (-0.20, 0.09) | -0.07 (-0.21, 0.07) | -0.07 (-0.21, 0.07) | -0.06 (-0.19, 0.08) |  |
| TGD | -0.29 (-1.8, 1.2) | -0.37 (-1.9, 1.2) | -0.5 (-2.1, 1.1) | -0.6 (-2.2, 0.98) | -0.61 (-2.2, 0.96) | -0.59 (-2.1, 0.90) |  |
| Sexual Orientation |  | | | | | |  |
| Bisexual | 0.65 (0.40, 0.90)*** | 0.73 (0.47, 1.0)*** | 0.73 (0.46, 1.0)*** | 0.56 (0.29, 0.83)*** | 0.52 (0.25, 0.79)*** | 0.52 (0.26, 0.77)*** |  |
| Gay or Lesbian | 0.70 (0.26, 1.1)** | 0.68 (0.22, 1.1)** | 0.69 (0.23, 1.2)** | 0.51 (0.05, 0.98)* | 0.50 (0.03, 0.96)* | 0.49 (0.05, 0.93)* |  |
| Additional Sexual Orientation | 0.64 (0.34, 0.93)*** | 0.61 (0.30, 0.92)*** | 0.53 (0.22, 0.85)*** | 0.40 (0.09, 0.71)* | 0.37 (0.06, 0.69)* | 0.40 (0.11, 0.69)** |  |
| Gender * Sexual Orientation |  | | | | | |  |
| Male * Bisexual | -0.03 (-0.80, 0.73) | -0.12 (-0.92, 0.67) | -0.19 (-1.0, 0.62) | -0.13 (-0.94, 0.68) | -0.11 (-0.92, 0.70) | -0.03 (-0.80, 0.73) |  |
| TGD * Bisexual | 0.75 (-1.1, 2.6) | 0.79 (-1.2, 2.8) | 1 (-0.97, 3.0) | 1 (-0.95, 3.0) | 1 (-0.95, 3.0) | 1.1 (-0.82, 2.9) |  |
| Male * Gay or Lesbian | -0.71 –(1.3, -0.13)* | -0.71 (-1.3, -0.11)* | -0.75 (-1.4, -0.14)* | -0.56 (-1.2, 0.05) | -0.56 (-1.2, 0.05) | -0.54 (-1.1, 0.04) |  |
| TGD * Gay or Lesbian | 0.51 (-1.6, 2.6) | 0.69 (-1.5, 2.9) | 0.48 (-1.7, 2.7) | 0.63 (-1.6, 2.8) | 0.63 (-1.6, 2.8) | 0.58 (-1.5, 2.7) |  |
| Male * Additional Sexual Orientation | -0.57 (-1.2, 0.08) | -0.55 (-1.2, 0.13) | -0.53 (-1.2, 0.16) | -0.47 (-1.2, 0.22) | -0.45 (-1.1, 0.23) | -0.49 (-1.1, 0.16) |  |
| TGD * Additional Sexual Orientation | 1.6 (-0.04, 3.2) | 1.7 (0.01, 3.4)* | 1.9 (0.20, 3.6)* | 2.0 (0.33, 3.8)* | 2.1 (0.34, 3.8)* | 2.0 (0.39, 3.6)* |  |
| Month * Gender |  | | | | | |  |
| Month * Male | -0.24 (-0.38, -0.09)** | -0.22 (-0.36, -0.07)** | -0.20 (-0.36, -0.05)* | -0.20 (-0.36, -0.05)* | -0.20 (-0.36, -0.05)* | -0.26 (-0.46, -0.06)** |  |
| Month * TGD | 0.67 (-0.96, 2.3) | 0.69 (-0.96, 2.3) | 0.42 (-1.4, 2.2) | 0.45 (-1.4, 2.3) | 0.45 (-1.4, 2.3) | 0.76 (-1.5, 3.0) |  |
| Month^2^ * Gender |  | | | | | |  |
| Month^2^ * Male | 0 (-0.05, 0.05) | -0.01 (-0.06, 0.04) | -0.01 (-0.06, 0.04) | -0.01 (-0.06, 0.04) | -0.01 (-0.06, 0.04) | 0.01 (-0.05, 0.07) |  |
| Month^2^ * TGD | 0.03 (-0.57, 0.62) | 0.02 (-0.58, 0.63) | 0.09 (-0.57, 0.75) | 0.08 (-0.58, 0.74) | 0.08 (-0.58, 0.74) | -0.09 (-0.87, 0.69) |  |
| Month * Sexual Orientation |  | | | | | |  |
| Month * Bisexual | 0.71 (0.44, 0.97)*** | 0.77 (0.50, 1.0)*** | 0.75 (0.46, 1.0)*** | 0.75 (0.46, 1.0)*** | 0.75 (0.46, 1.0)*** | 0.72 (0.36, 1.1)*** |  |
| Month * Gay or Lesbian | -0.09 (-0.57, 0.38) | -0.09 (-0.57, 0.39) | -0.04 (-0.56, 0.48) | -0.04 (-0.57, 0.48) | -0.04 (-0.57, 0.48) | -0.06 (-0.71, 0.58) |  |
| Month * Additional Sexual Orientation | 0.80 (0.50, 1.1)*** | 0.82 (0.52, 1.1)*** | 0.65 (0.32, 0.99)*** | 0.65 (0.32, 0.98)*** | 0.65 (0.32, 0.98)*** | 0.66 (0.24, 1.1)** |  |
| Month^2^ * Sexual Orientation |  | | | | | |  |
| Month^2^ * Bisexual | -0.22 (0.31, -0.14)*** | -0.24 (-0.33, -0.16)*** | -0.24 (-0.33, -0.15)*** | -0.24 (-0.33, -0.15)*** | -0.24 (-0.33, -0.15)*** | -0.23 (-0.34, -0.11)*** |  |
| Month^2^ * Gay or Lesbian | 0 (-0.17, 0.16) | -0.01 (-0.17, 0.16) | -0.01 (-0.19, 0.16) | -0.01 (-0.19, 0.16) | -0.01 (-0.19, 0.16) | -0.03 (-0.24, 0.18) |  |
| Month^2^ * Additional Sexual Orientation | -0.25 (-0.35, -0.15)*** | -0.25 (-0.35, -0.15)*** | -0.20 (-0.30, -0.09)*** | -0.20 (-0.30, -0.09)*** | -0.20 (-0.30, -0.09)*** | -0.20 (-0.33, -0.07)** |  |
| Month * Gender * Sexual Orientation |  | | | | | |  |
| Month * Male * Bisexual | -0.05 (-0.89, 0.80) | -0.07 (-0.92, 0.78) | -0.2 (-1.1, 0.75) | -0.2 (-1.1, 0.75) | -0.2 (-1.1, 0.75) | -0.17 (-1.3, 0.99) |  |
| Month * TGD * Bisexual | -1.4 (-3.4, 0.65) | -1.3 (-3.4, 0.70) | -0.84 (-3.1, 1.4) | -0.88 (-3.1, 1.3) | -0.88 (-3.1, 1.3) | -0.88 (-3.6, 1.9) |  |
| Month * Male * Gay or Lesbian | 0.6 (-0.02, 1.2) | 0.62 (-0.01, 1.2) | 0.54 (-0.15, 1.2) | 0.54 (-0.14, 1.2) | 0.54 (-0.14, 1.2) | 0.54 (-0.31, 1.4) |  |
| Month * TGD * Gay or Lesbian | -0.62 (-2.8, 1.6) | -0.76 (-3.0, 1.4) | -1.2 (-3.6, 1.2) | -1.2 (-3.6, 1.2) | -1.2 (-3.6, 1.2) | -1.4 (-4.4, 1.6) |  |
| Month * Male * Additional Sexual Orientation | -0.27 (-0.96, 0.41) | -0.23 (-0.92, 0.47) | -0.2 (-0.95, 0.56) | -0.19 (-0.95, 0.56) | -0.19 (-0.95, 0.56) | -0.15 (-1.1, 0.79) |  |
| Month * TGD * Additional Sexual Orientation | -1.1 (-2.8, 0.70) | -1.1 (-2.9, 0.68) | -0.71 (-2.7, 1.2) | -0.74 (-2.7, 1.2) | -0.74 (-2.7, 1.2) | -1.1 (-3.5, 1.3) |  |
| Month^2^ * Gender * Sexual Orientation |  | | | | | |  |
| Month^2^ * Male * Bisexual | 0.02 (-0.26, 0.30) | 0.04 (-0.24, 0.32) | 0.07 (-0.24, 0.38) | 0.07 (-0.24, 0.38) | 0.07 (-0.24, 0.38) | 0.05 (-0.31, 0.42) |  |
| Month^2^ * TGD * Bisexual | 0.4 (-0.31, 1.1) | 0.39 (-0.33, 1.1) | 0.27 (-0.51, 1.1) | 0.28 (-0.50, 1.1) | 0.28 (-0.50, 1.1) | 0.27 (-0.66, 1.2) |  |
| Month^2^ * Male * Gay or Lesbian | -0.14 (-0.35, 0.07) | -0.14 (-0.35, 0.07) | -0.12 (-0.35, 0.11) | -0.12 (-0.35, 0.10) | -0.12 (-0.35, 0.10) | -0.08 (-0.35, 0.19) |  |
| Month^2^ * TGD * Gay or Lesbian | 0.02 (-0.73, 0.76) | 0.06 (-0.70, 0.81) | 0.19 (-0.62, 1.0) | 0.2 (-0.61, 1.0) | 0.2 (-0.61, 1.0) | 0.32 (-0.68, 1.3) |  |
| Month^2^ * Male * Additional Sexual Orientation | 0.14 (-0.09, 0.36) | 0.12 (-0.11, 0.35) | 0.11 (-0.14, 0.36) | 0.11 (-0.14, 0.35) | 0.11 (-0.14, 0.35) | 0.11 (-0.19, 0.41) |  |
| Month^2^ * TGD * Additional Sexual Orientation | 0.08 (-0.55, 0.71) | 0.09 (-0.55, 0.73) | -0.01 (-0.71, 0.69) | -0.01 (-0.70, 0.69) | -0.01 (-0.70, 0.69) | 0.19 (-0.64, 1.0) |  |
| Session Last 7 Days |  | -0.86 (-0.91, -0.80)*** | -1.0 (-1.1, -0.94)*** | -1.0 (-1.1, -0.95)*** | -1.0 (-1.1, -0.95)*** | -0.99 (-1.0, -0.94)*** |  |
| Gender * Session Last 7 Days |  | | | | | |  |
| Male * Session Last 7 Days |  | 0 (-0.09, 0.09) | 0.01 (-0.08, 0.11) | 0.02 (-0.08, 0.11) | 0.02 (-0.08, 0.11) | 0.01 (-0.08, 0.10) |  |
| TGD * Session Last 7 Days |  | 0.12 (-1.0, 1.3) | 0.16 (-1.0, 1.3) | 0.11 (-1.0, 1.3) | 0.11 (-1.0, 1.3) | 0.06 (-1.1, 1.2) |  |
| Sexual Orientation * Session Last 7 Days |  | | | | | |  |
| Bisexual * Session Last 7 Days |  | -0.21 (-0.38, -0.04)* | -0.21 (-0.38, -0.04)* | -0.21 (-0.39, -0.04)* | -0.21 (-0.39, -0.04)* | -0.20 (-0.37, -0.03)* |  |
| Gay or Lesbian * Session Last 7 Days |  | 0.04 (-0.28, 0.35) | 0.03 (-0.29, 0.34) | 0.03 (-0.29, 0.34) | 0.03 (-0.29, 0.34) | 0.01 (-0.30, 0.32) |  |
| Additional Sexual Orientation * Session Last 7 Days |  | 0.06 (-0.14, 0.25) | 0.1 (-0.10, 0.30) | 0.11 (-0.09, 0.30) | 0.11 (-0.09, 0.30) | 0.09 (-0.11, 0.28) |  |
| Gender * Sexual Orientation * Session Last 7 Days |  | | | | | |  |
| Male * Bisexual * Session Last 7 Days |  | 0.2 (-0.32, 0.72) | 0.2 (-0.33, 0.73) | 0.2 (-0.32, 0.73) | 0.2 (-0.32, 0.73) | 0.12 (-0.39, 0.63) |  |
| TGD * Bisexual * Session Last 7 Days |  | -0.01 (-1.4, 1.3) | -0.11 (-1.5, 1.3) | -0.06 (-1.4, 1.3) | -0.06 (-1.4, 1.3) | -0.13 (-1.5, 1.2) |  |
| Male * Gay or Lesbian * Session Last 7 Days |  | -0.04 (-0.45, 0.37) | -0.01 (-0.42, 0.40) | -0.01 (-0.42, 0.40) | -0.01 (-0.42, 0.40) | 0 (-0.40, 0.40) |  |
| TGD * Gay or Lesbian * Session Last 7 Days |  | -0.3 (-1.8, 1.2) | -0.16 (-1.7, 1.4) | -0.11 (-1.6, 1.4) | -0.11 (-1.6, 1.4) | 0.1 (-1.4, 1.6) |  |
| Male * Additional Sexual Orientation * Session Last 7 Days |  | -0.04 (-0.48, 0.39) | -0.07 (-0.51, 0.37) | -0.06 (-0.50, 0.38) | -0.06 (-0.50, 0.38) | -0.05 (-0.48, 0.37) |  |
| TGD * Additional Sexual Orientation * Session Last 7 Days |  | -0.18 (-1.4, 1.0) | -0.27 (-1.5, 0.97) | -0.23 (-1.5, 1.0) | -0.23 (-1.5, 1.0) | -0.16 (-1.4, 1.0) |  |
| Session Last 8-14 Days |  | | -0.86 (-0.92, -0.81)*** | -0.86 (-0.92, -0.81)*** | -0.86 (-0.92, -0.81)*** | -0.81 (-0.86, -0.75)*** |  |
| Gender * Session Last 8-14 Days |  | | | | | |  |
| Male * Session Last 8-14 Days |  | | 0.02 (-0.08, 0.11) | 0.02 (-0.08, 0.11) | 0.02 (-0.08, 0.11) | 0.01 (-0.08, 0.11) |  |
| TGD * Session Last 8-14 Days |  | | 0.4 (-0.78, 1.6) | 0.37 (-0.81, 1.6) | 0.37 (-0.81, 1.6) | 0.16 (-1.0, 1.3) |  |
| Sexual Orientation * Session Last 8-14 Days |  | | | | | |  |
| Bisexual * Session Last 8-14 Days |  | | 0.01 (-0.16, 0.19) | 0.02 (-0.16, 0.19) | 0.02 (-0.16, 0.19) | 0.04 (-0.13, 0.21) |  |
| Gay or Lesbian * Session Last 8-14 Days |  | | -0.05 (-0.37, 0.27) | -0.05 (-0.37, 0.27) | -0.05 (-0.37, 0.27) | 0.01 (-0.31, 0.32) |  |
| Additional Sexual Orientation * Session Last 8-14 Days |  | | 0.25 (0.04, 0.45)* | 0.25 (0.05, 0.45)* | 0.25 (0.05, 0.45)* | 0.21 (0.01, 0.41)* |  |
| Gender * Sexual Orientation * Session Last 8-14 Days |  | | | | | |  |
| Male * Bisexual * Session Last 8-14 Days |  | | 0.26 (-0.30, 0.82) | 0.26 (-0.30, 0.82) | 0.26 (-0.30, 0.82) | 0.09 (-0.46, 0.64) |  |
| TGD * Bisexual * Session Last 8-14 Days |  | | -0.78 (-2.2, 0.61) | -0.76 (-2.2, 0.64) | -0.76 (-2.2, 0.64) | -0.62 (-2.0, 0.75) |  |
| Male * Gay or Lesbian * Session Last 8-14 Days |  | | 0.09 (-0.33, 0.51) | 0.09 (-0.33, 0.51) | 0.09 (-0.33, 0.51) | -0.01 (-0.42, 0.40) |  |
| TGD * Gay or Lesbian * Session Last 8-14 Days |  | | 0.69 (-0.86, 2.2) | 0.72 (-0.83, 2.3) | 0.72 (-0.83, 2.3) | 0.82 (-0.71, 2.3) |  |
| Male * Additional Sexual Orientation * Session Last 8-14 Days |  | | -0.01 (-0.45, 0.43) | -0.01 (-0.45, 0.43) | -0.01 (-0.45, 0.43) | -0.02 (-0.44, 0.41) |  |
| TGD * Additional Sexual Orientation * Session Last 8-14 Days |  | | -0.64 (-1.9, 0.62) | -0.62 (-1.9, 0.64) | -0.62 (-1.9, 0.64) | -0.39 (-1.6, 0.85) |  |
| Educational Attainment |  | | | | | |  |
| Missing or Unknown |  | | | 0.41 (0.03, 0.78)* | 0.39 (0.01, 0.77)* | 0.39 (0.03, 0.75)* |  |
| Not college graduate |  | | | 0.96 (0.84, 1.1)*** | 0.96 (0.84, 1.1)*** | 0.97 (0.86, 1.1)*** |  |
| Age (Years, Mean Centered) |  | | | | -0.01 (-0.01, 0.00)** | -0.01 (-0.01, 0.00) |  |
| **Model Selection Metrics** | | | | | | |  |
| AIC | 672,998 | 671,248 | 669,666 | 669,422 | 669,416 | 665,267 |  |
| BIC | 673,396 | 671,762 | 670,297 | 670,071 | 670,075 | 665,955 |  |
| Log Likelihood | -336,458 | -335,571 | -334,768 | -334,644 | -334,640 | -332,563 |  |
| Chi-Square Difference Test (*P* Value) | -- | 1,774 (<0.001) | 1,606 (<0.001) | 249 (<0.001) | 8 (0.004) | 4,155 (<0.001) |  |

**Notes.** PHQ: 9-item Patient Health Questionnaire. Reference category: Straight, female, college graduate or above. Analysis only included participants with baseline PHQ-9 ≥ 10

^a^Model 1: Month, month^2^, gender identity, sexual orientation, gender identity * sexual orientation, month * gender identity,month^2^ * gender identity, month * sexual orientation, month^2^ * sexual orientation, month * gender identity * sexual orientation, month^2^* gender identity * sexual orientation, random effects for provider and patient nested under provider, random effect for month per patient nested under provider

^b^Model 2:  Model 1 + session last 7 days, session last 7 days * gender identity, session last 7 days * sexual orientation,  session last 7 days * gender identity * sexual orientation

^c^Model 3: Model 2 + session last 8-14 days, session last 8-14 days* gender identity,  session last 8-14 days * sexual orientation, session last 8-14 days * gender identity * sexual orientation

^d^Model 4: Model 3 + education

^e^Model 5: Model 4 + age (grand mean centered)

^f^Model 6: Model 5 + additional random effect term for month^2^ per clients nested under provider

*p<0.05; **p<0.01; ***p<0.001

**Table S5.** Post-hoc tests: Rates of reliable improvement and/or recovery in symptoms of anxiety (GAD-7) or depression (PHQ-9) across gender identity and sexual orientation group

| **(a) Reliable Improvement across gender identity group^a^** | | | | | | |
| --- | --- | --- | --- | --- | --- | --- |
| **Dimension** | **Value** | **Female** | **Male** | **TGD** | **Unknown** |  |
| No reliable improvement | Residuals | 1.77 | -2.81 | 1.49 | 1.53 |  |
|  | *P* value | 0.61 | 0.04 | >0.99 | >0.99 |  |
| Reliable improvement | Residuals | -1.77 | 2.81 | -1.49 | -1.53 |  |
|  | *P* value | 0.61 | 0.04 | >0.99 | >0.99 |  |
| **(b) Reliable Improvement across sexual orientation group^a^** | | | | | | |
| **Dimension** | **Value** | **Bisexual** | **Gay or Lesbian** | **Additional Sexual Orientation** | **Straight** | **Unknown** |
| No reliable improvement | Residuals | 2.09 | 0.17 | 2.75 | -4.10 | 2.21 |
|  | *P* value | 0.37 | >0.99 | 0.06 | <0.001 | 0.27 |
| Reliable improvement | Residuals | -2.09 | -0.17 | -2.75 | 4.10 | -2.21 |
|  | *P* value | 0.37 | >0.99 | 0.06 | <0.001 | 0.27 |
| (**c) Recovery across gender identity group^b^** | | | | | | |
| **Dimension** | **Value** | **Female** | **Male** | **TGD** | **Unknown** |  |
| No Recovery | Residuals | 4.16 | -5.58 | 3.41 | 1.05 |  |
| No Recovery | *P* value | <0.001 | <0.001 | 0.005 | >0.99 |  |
| Recovery | Residuals | -4.16 | 5.58 | -3.41 | -1.05 |  |
| Recovery | *P* value | <0.001 | <0.001 | 0.005 | >0.99 |  |
| **(d) Recovery across sexual orientation group**^b^ | | | | | | |
| **Dimension** | **Value** | **Bisexual** | **Gay or Lesbian** | **Additional Sexual Orientation** | **Straight** | **Unknown** |
| No Recovery | Residuals | 4.65 | 1.14 | 2.92 | -6.14 | 2.34 |
| No Recovery | *P* value | <0.001 | >0.99 | 0.04 | <0.001 | 0.19 |
| Recovery | Residuals | -4.65 | -1.14 | -2.92 | 6.14 | -2.34 |
| Recovery | *P* value | <0.001 | >0.99 | 0.04 | <0.001 | 0.19 |
| **(e) Reliable Improvement AND recovery across gender identity group^c^** | | | | | | |
| **Dimension** | **Value** | **Female** | **Male** | **TGD** | **Unknown** |  |
| No Reliable Improvement and recovery | Residuals | 3.84 | -5.03 | 2.56 | 1.04 |  |
| No Reliable Improvement and recovery | *P* value | <0.001 | <0.001 | 0.08 | >0.99 |  |
| Reliable Improvement and recovery | Residuals | -3.84 | 5.03 | -2.56 | -1.04 |  |
| Reliable Improvement and recovery | *P* value | <0.001 | <0.001 | 0.08 | >0.99 |  |
| **(f) Reliable improvement AND recovery across sexual orientation group^c^** | | | | | | |
| **Dimension** | **Value** | **Bisexual** | **Gay or Lesbian** | **Additional  Sexual Orientation** | **Straight** | **Unknown** |
| No Reliable Improvement and recovery | Residuals | 4.28 | 1.39 | 2.97 | -6.51 | 3.04 |
| No Reliable Improvement and recovery | *P* value | <0.001 | >0.99 | 0.03 | <0.001 | 0.02 |
| Reliable Improvement and recovery | Residuals | -4.28 | -1.39 | -2.97 | 6.51 | -3.04 |
| Reliable Improvement and recovery | *P* value | <0.001 | >0.99 | 0.03 | <0.001 | 0.02 |
| **(g) Reliable Improvement OR recovery across gender identity group^d^** | | | | | | |
| **Dimension** | **Value** | **Female** | **Male** | **TGD** | **Unknown** |  |
| No Reliable Improvement or recovery | Residuals | 2.23 | -3.61 | 2.43 | 1.75 |  |
| No Reliable Improvement or recovery | *P* value | 0.20 | 0.002 | 0.12 | 0.64 |  |
| Reliable Improvement or recovery | Residuals | -2.23 | 3.61 | -2.43 | -1.75 |  |
| Reliable Improvement or recovery | *P* value | 0.20 | 0.002 | 0.12 | 0.64 |  |
| **(h) Reliable improvement OR recovery across sexual orientation group^d^** | | | | | | |
| **Dimension** | **Value** | **Bisexual** | **Gay or Lesbian** | **Additional Sexual Orientation** | **Straight** | **Unknown** |
| No Reliable Improvement or recovery | Residuals | 2.49 | -0.09 | 2.62 | -3.82 | 1.74 |
| No Reliable Improvement or recovery | *P* value | 0.13 | >0.99 | 0.09 | 0.001 | 0.82 |
| Reliable Improvement or recovery | Residuals | -2.49 | 0.09 | -2.62 | 3.82 | -1.74 |
| Reliable Improvement or recovery | *P* value | 0.13 | >0.99 | 0.09 | 0.001 | 0.82 |

**Notes**. TGD: Transgender and gender diverse. GAD-7: 7-item Generalized Anxiety Disorder Scale. PHQ: 9-item Patient Health Questionnaire.

^a^Reliable improvement: ≥ 4 point decrease on the final GAD-7 among those with baseline GAD-7 ≥ 8; and/or ≥ 6 point decrease on the final PHQ-9 among those with baseline PHQ-9 ≥ 10

^b^Recovery: Final GAD-7 < 8 among those with baseline GAD-7 ≥ 8; and/or final PHQ-9 < 10 among those with baseline PHQ-9 ≥ 10

^c^Reliable Improvement and Recovery: ≥ 4 point decrease on the final GAD-7 and final GAD-7 < 8 among those with baseline GAD-7 ≥ 8; and/or  ≥ 6 point decrease on the final PHQ-9 and final PHQ-9 < 10 among those with baseline PHQ-9 ≥ 10

^d^Reliable Improvement or Recovery: ≥ 4 point decrease on the final GAD-7 or final GAD-7 < 8 among those with baseline GAD-7 ≥ 8; and/or  ≥ 6 point decrease on the final PHQ-9 or final PHQ-9 < 10 among those with baseline PHQ-9 ≥ 10.

**Table S6.** Post-hoc tests: Pairwise Differences in treatment duration and satisfaction scores across gender identity and sexual orientation group

| **Pairwise comparisons of session count across gender identity group** | | | |
| --- | --- | --- | --- |
| **Comparison** | **Z** | **Unadjusted p-value** | **Adjusted *P* value^a^** |
| Female - Male | 0.93 | 0.35 | >0.99 |
| Female - TGD | 0.37 | 0.71 | >0.99 |
| Male - TGD | 0.13 | 0.89 | >0.99 |
| Female - Unknown | 3.29 | 0.001 | 0.006 |
| Male - Unknown | 2.85 | 0.004 | 0.03 |
| TGD - Unknown | 1.50 | 0.13 | 0.81 |
| **Pairwise comparisons of session count across sexual orientation group** | | | |
| **Comparison** | **Z** | **Unadjusted p-value** | **Adjusted *P* value^a^** |
| Bisexual - Gay or Lesbian | 1.23 | 0.22 | >0.99 |
| Bisexual - Additional Sexual Orientation | -0.72 | 0.47 | >0.99 |
| Gay or Lesbian - Additional Sexual Orientation | -1.89 | 0.06 | 0.59 |
| Bisexual - Straight | 0.91 | 0.36 | >0.99 |
| Gay or Lesbian - Straight | -0.78 | 0.43 | >0.99 |
| Additional Sexual Orientation - Straight | 1.88 | 0.06 | 0.59 |
| Bisexual - Unknown | 2.66 | 0.01 | 0.08 |
| Gay or Lesbian - Unknown | 1.15 | 0.25 | >0.99 |
| Additional Sexual Orientation - Unknown | 3.40 | <0.001 | 0.01 |
| Straight - Unknown | 2.82 | 0.005 | 0.05 |
| **Pairwise comparisons of treatment duration across gender identity group** | | | |
| **Comparison** | **Z** | **Unadjusted p-value** | **Adjusted *P* value^a^** |
| Female - Male | 2.10 | 0.04 | 0.22 |
| Female - TGD | 0.50 | 0.62 | >0.99 |
| Male - TGD | -0.03 | 0.97 | >0.99 |
| Female - Unknown | 3.76 | <0.001 | 0.001 |
| Male - Unknown | 2.86 | 0.004 | 0.03 |
| TGD - Unknown | 1.65 | 0.10 | 0.60 |
| **Pairwise comparisons of treatment duration across sexual orientation group** | | | |
| **Comparison** | **Z** | **Unadjusted p-value** | **Adjusted *P* value^a^** |
| Bisexual - Gay or Lesbian | 0.68 | 0.50 | >0.99 |
| Bisexual - Additional Sexual Orientation | -0.34 | 0.73 | >0.99 |
| Gay or Lesbian - Additional Sexual Orientation | -0.99 | 0.32 | >0.99 |
| Bisexual - Straight | 0.04 | 0.97 | >0.99 |
| Gay or Lesbian - Straight | -0.84 | 0.40 | >0.99 |
| Additional Sexual Orientation - Straight | 0.51 | 0.61 | >0.99 |
| Bisexual - Unknown | 2.36 | 0.02 | 0.18 |
| Gay or Lesbian - Unknown | 1.46 | 0.15 | >0.99 |
| Additional Sexual Orientation - Unknown | 2.71 | 0.01 | 0.07 |
| Straight - Unknown | 3.37 | <0.001 | 0.01 |
| **Pairwise comparisons of treatment satisfaction across gender identity group** | | | |
| **Comparison** | **Z** | **Unadjusted p-value** | **Adjusted *P* value^a^** |
| Female - Male | 4.78 | <0.001 | <0.001 |
| Female - TGD | 2.74 | 0.01 | 0.04 |
| Male - TGD | 1.44 | 0.15 | 0.91 |
| Female - Unknown | 2.08 | 0.04 | 0.22 |
| Male - Unknown | 0.21 | 0.84 | >0.99 |
| TGD - Unknown | -1.10 | 0.27 | >0.99 |
| **Pairwise comparisons of treatment satisfaction across sexual orientation group** | | | |
| **Comparison** | **Z** | **Unadjusted p-value** | **Adjusted *P* value^a^** |
| Bisexual - Gay or Lesbian | 0.54 | 0.59 | >0.99 |
| Bisexual - Additional Sexual Orientation | 0.61 | 0.54 | >0.99 |
| Gay or Lesbian - Additional Sexual Orientation | 0.02 | 0.99 | >0.99 |
| Bisexual - Straight | 1.06 | 0.29 | >0.99 |
| Gay or Lesbian - Straight | 0.21 | 0.83 | >0.99 |
| Additional Sexual Orientation - Straight | 0.23 | 0.82 | >0.99 |
| Bisexual - Unknown | 2.92 | 0.003 | 0.03 |
| Gay or Lesbian - Unknown | 2.06 | 0.04 | 0.39 |
| Additional Sexual Orientation - Unknown | 2.28 | 0.02 | 0.23 |
| Straight - Unknown | 3.04 | 0.002 | 0.02 |

**Notes**.  TGD: Transgender and gender diverse. ^a^Adjusted p-value: denotes the Bonferroni-adjusted p-values.
